# Concerted Action of Two Formins in Gliding Motility and Host Cell Invasion by *Toxoplasma gondii*

**DOI:** 10.1371/journal.ppat.1001132

**Published:** 2010-10-07

**Authors:** Wassim Daher, Fabienne Plattner, Marie-France Carlier, Dominique Soldati-Favre

**Affiliations:** 1 Department of Microbiology and Molecular Medicine, CMU, University of Geneva, Geneva, Switzerland; 2 Dynamique du Cytosquelette, Laboratoire d'Enzymologie et Biochimie Structurales UPR A 9063, CNRS, Gif sur Yvette, France; University of Michigan, United States of America

## Abstract

The invasive forms of apicomplexan parasites share a conserved form of gliding motility that powers parasite migration across biological barriers, host cell invasion and egress from infected cells. Previous studies have established that the duration and direction of gliding motility are determined by actin polymerization; however, regulators of actin dynamics in apicomplexans remain poorly characterized. In the absence of a complete ARP2/3 complex, the formin homology 2 domain containing proteins and the accessory protein profilin are presumed to orchestrate actin polymerization during host cell invasion. Here, we have undertaken the biochemical and functional characterization of two *Toxoplasma gondii* formins and established that they act in concert as actin nucleators during invasion. The importance of TgFRM1 for parasite motility has been assessed by conditional gene disruption. The contribution of each formin individually and jointly was revealed by an approach based upon the expression of dominant mutants with modified FH2 domains impaired in actin binding but still able to dimerize with their respective endogenous formin. These mutated FH2 domains were fused to the ligand-controlled destabilization domain (DD-FKBP) to achieve conditional expression. This strategy proved unique in identifying the non-redundant and critical roles of both formins in invasion. These findings provide new insights into how controlled actin polymerization drives the directional movement required for productive penetration of parasites into host cells.

## Author Summary

Gliding motility is a unique property of the Apicomplexa. Members of this phylum include important human and animal pathogens. An actomyosin-based machine powers parasite motility and is crucial for parasite migration across biological barriers, host cell invasion and egress from infected cells. The timing, duration and orientation of the gliding motility are tightly regulated to insure successful establishment of infection. Controlled polymerization of actin filaments is a key feature of motility, and we demonstrate here the implication of two formins that catalyse actin nucleation and fast assembly of filaments. Both proteins are essential and act in concert during productive penetration of the parasite into host cells.

## Introduction

The phylum Apicomplexa encompasses pathogens of significant medical relevance including those responsible for malaria and toxoplasmosis. These parasites cross biological barriers and enter cells by an active process that depends on a unique form of gliding motility [1]. In *Toxoplasma gondii*, drugs that interfere with actin assembly and dynamics have revealed that gliding critically relies on an intact parasite actin cytoskeleton [2] and requires actin polymerization [3]. Moreover, previous work using reverse genetics highlighted that gliding is powered by the myosin XIV, TgMyoA [4].

Paradoxically, visualisation of actin filaments under physiological conditions, either by electron microscopy or by staining with phalloidin, has proven very difficult in the phylum. Sedimentation experiments suggested that actin is maintained in a globular form (>98%) [5]. Work performed *in vitro* on purified or recombinant actins revealed that preferentially short (0.1 µm) actin filaments are assembled [6], [7], [8], hence actin might be tailored to undergo rapid cycles of assembly and disassembly. Among the systems orchestrating actin nucleation, the Arp2/3 complex, generates a network of short, branched filaments, whereas the formin-profilin system catalyzes the processive assembly of unbranched actin filaments [9]. The Apicomplexans lack many actin-regulatory proteins including the Arp2/3 complex [10]. In contrast, they contain at least two formins and a profilin that have been previously associated with parasite motility [11], [12], [13].

Formins constitute a large family of proteins involved in many biological processes including cell polarity, cell-cell contact, cell and tissue morphogenesis, cytokinesis, filopodia formation, stress fiber formation, motility and in microtubule-actin cross talk to maintain the cell cytoskeleton [14]. These proteins are composed of multi-domains interacting with other cellular factors to promote actin nucleation and polymerization. The common feature of all formins is the FH2 domain, which nucleates actin assembly and binds the barbed end at nanomolar concentrations allowing the formation of linear and unbranched actin filaments [15], [16].

The second domain catalyzing the activity of formins is the FH1 for “formin homology 1 domain”. FH1 is typically positioned immediately N-terminal to the FH2 domain and is composed of poly-proline stretches that bind specifically to the profilin-actin complex during barbed end filament elongation [14]. The FH2 domain associates with the barbed end (fast growing plus end of actin filament) of actin filaments and in association with the FH1 domain promotes rapid processive barbed end assembly from profilin-actin, increasing the association rate constant of profilin-actin to barbed ends by 2 to 15 fold [14]. Profilin-actin is involved in a rapid delivery step by which FH1-profilin-actin is transferred directly to the FH2-associated barbed end [9]. Formin activity is frequently regulated by autoinhibition, which is maintained by the binding of the C-terminal diaphanous autoregulatory domain (DAD) segment to the diaphanous inhibitory domain (DID). Binding of Rho to the GTPase-binding domain (GBD) releases the autoinhibition to activate formin [9].

In *T. gondii,* TgPRF participates in barbed end growth and a conditional knockdown of the gene established its vital role in motility and invasion [13]. *P. falciparum* formins 1 and 2 (PfFRM1 and PfFRM2) nucleate chicken actin polymerization *in vitro* [12] and localization of PfFRM1 at the site of contact between invading merozoites and the host cell suggested a role in invasion [12]. Recently, a formin-like protein termed MISFIT was localized to the nucleus of male gametocytes, zygotes and ookinetes of the rodent malaria parasite *Plasmodium berghei.* This protein was shown to regulate cell cycle progression from ookinete to oocysts but an activity of actin nucleation has not been reported [17]. The *T. gondii* genome does not contain a gene coding for MISFIT but instead encodes a third putative formin (TgFRM3) that is only found in coccidians and whose function is dispensable and not linked to motility and invasion (Daher, unpublished).

In this study, we developed a genetic strategy based upon the expression of dominant negative mutants to establish that both TgFRM1 and TgFRM2 contribute to motility and invasion. This approach was validated by biochemical analyses, which highlighted the distinct properties of the two formins. TgFRM1 and TgFRM2 promote and control growth of the filaments and possibly stabilize their position and directionality to drive parasite entry into host cells.

## Results

### TgFRM1 and TgFRM2 are differentially localized to the pellicle

**Figure 1:**
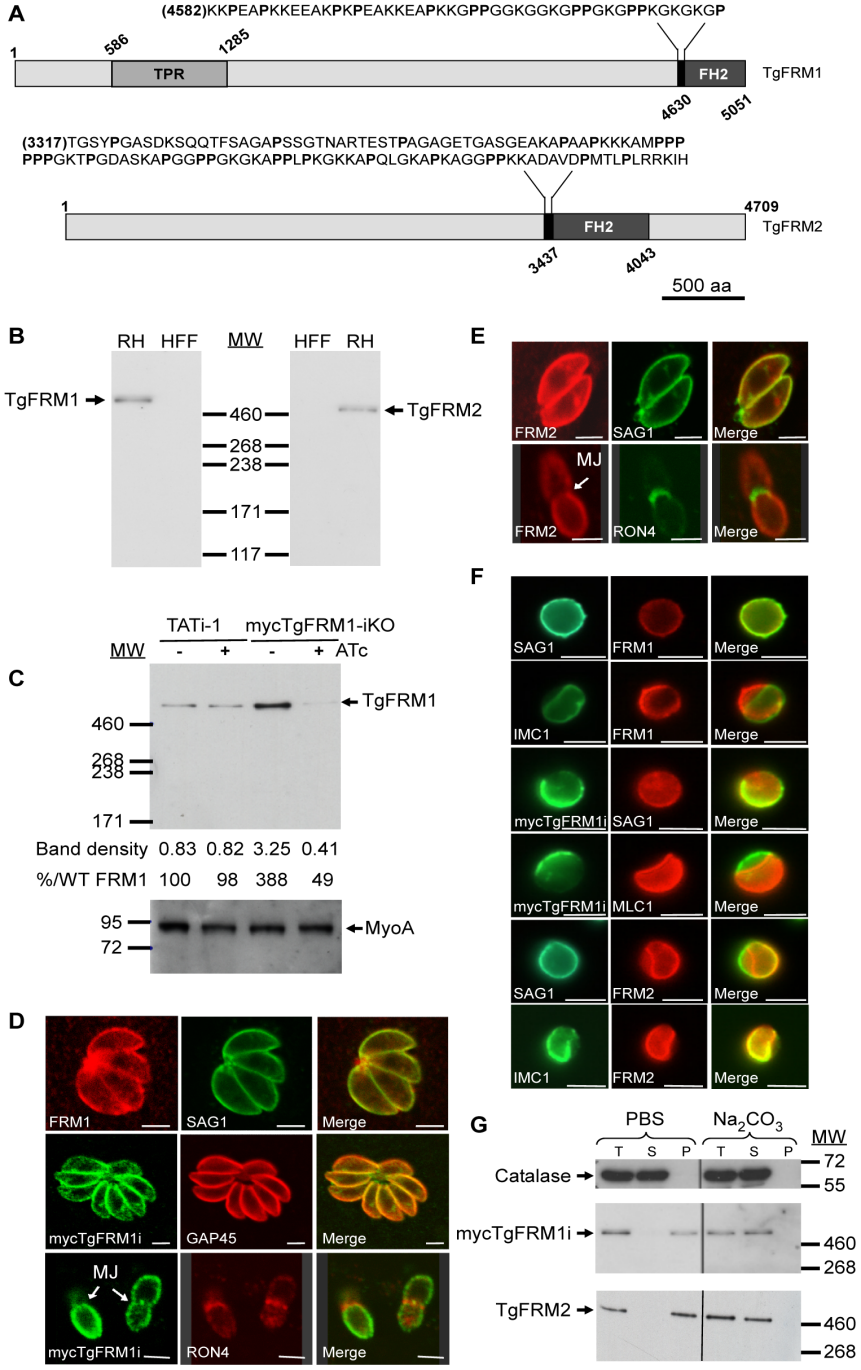
Expression and localization of TgFRM1 and TgFRM2 in tachyzoites. (A) Schematic representation of *Toxoplasma gondii* formin 1 (TgFRM1) and formin 2 (TgFRM2) showing the domains of homolgy to tetratricopeptide repeat (TPR) and the formin homology 2 domain (FH2). Expanded sequences above protein schematic represent the potential formins 1 and 2 FH1 domains. The proline (P) residues are bolded. Numbers indicate amino acid residues of the different domains. Scale bar represents 500 aa. (B) Western blot analysis of parasite lysates (RHΔhxgprt, a type I virulent strain) and HFF (Human Foreskin Fibroblasts) cells probed with rabbit antisera specific to TgFRM1 and TgFRM2. (C) Western blot analysis of TATi-1 and MycTgFRM1-iKO strains in presence or in absence of ATc. Parasites were treated 4 days ± ATc. Myosin A (MyoA) served as loading control. The integrated densities of the bands measured using the ImageJ program, and the percentage of expression relative to TATi-1 in absence of ATc are provided. (D) The peripheral localization of TgFRM1 was determined by IFA using anti-TgFRM1 on RH and anti-myc on mycTgFRM1-iKO. SAG1 and GAP45 are used as markers of PM and IMC, respectively. RON4 is a marker of the moving junction (MJ). (E) TgFRM2 localized to the periphery of intracellular and extracellular parasites. Scale bars represent 2 µm. (F) IFA after aerolysin treatment. TgFRM1 and mycTgFRM1i colocalized with SAG1 at the PM, whereas TgFRM2 colocalized with IMC1. (G) Comparison of mycTgFRM1i and TgFRM2 solubility in PBS buffer and in PBS/Na_2_CO_3_ 0.1 M pH 11.5. Total lysate (T), the supernatant (S) and pellet (P) after fractionation.

BLASTP analysis of the ToxoDB database (http://www.ToxoDB.org) revealed the presence of three FH2 domain-containing proteins in *T. gondii* with two of them, TgFRM1 and TgFRM2 being conserved across the Apicomplexa phylum [12]. TgFRM1 and TgFRM2 are large proteins with a predicted molecular weight of 552 kDa and 492 kDa, respectively. The FH2 domain is positioned at the extreme carboxy-terminus in case of TgFRM1. The presence of a canonical FH1 is not apparent but both formins possess a segment rich in proline residues upstream of the FH2 (Figure 1A). These formins also lack the DAD and DID regulatory domains, however, TgFRM1 possesses four tetratricopeptide repeats also present on PfFRM1 (Figure 1A). Specific antibodies were raised against bacterially produced FH2 domains of both *T. gondii* formins and western blot analysis confirmed that TgFRM1 and TgFRM2 are expressed in tachyzoites and migrate at their predicted sizes on SDS-PAGE (Figure 1B). Parasite lines expressing the FH2 domains of either TgFRM1 or TgFRM2 were used to confirm the specificity and the absence of cross-reactivity of the two rabbit anti-sera (Figure S1B, S1C and S1D in Supporting Information S1).

To investigate the function and importance of these formins, conditional knockdowns were attempted in the TATi-1 strain, using the tetracycline-based transactivator system previously developed for *T. gondii* [4]. Given the unmanageable size of the *TgFRM1 and TgFRM2* cDNAs, we opted for a promoter exchange approach instead of the two step knockout strategy that requires the integration of a second inducible copy (Figure S2A in Supporting Information S1). To select for the recombinant lines of interest, a YFP expression cassette was inserted into the knockout vectors allowing FACS sorting of the parasites that underwent double homologous recombination (YFP negative) [18]. The *TgFRM1* promoter was replaced by the inducible *tetO7Sag4* and a myc-epitope tag was inserted at the N-terminus of the *TgFRM1* gene. Numerous attempts to replace the promoter of *TgFRM2* failed (18 transfections) and resulted only in a single homologous recombination event at the locus (data not shown). In contrast, positive mycTgFRM1-iKO (Table S2 in Supporting Information S1) clones were identified by an indirect immunofluorescence assay (IFA) and confirmed by genomic PCR (Figure S2B in Supporting Information S1). Western blot analysis using anti-FRM1 antibodies revealed that the level of mycTgFRM1i was considerably higher (ca. 4 fold) than the endogenous level of TgFRM1 in TATi-1 strain (Figure 1C). In the presence of anhydrotetracycline (ATc), mycTgFRM1i was significantly down-regulated to less than 15% but that still corresponded to ca. 45 to 50% of the endogenous level of TgFRM1 (Figure 1C; Figure S2C in Supporting Information S1). Both TgFRM1 and mycTgFRM1i localized to the periphery of replicating (intracellular) as well as invading (extracellular) parasites (Figure 1D), In contrast with a previous report [12], TgFRM1 was not found to be selectively redistributed to the apical pole, but this discrepancy may be explained by different fixation protocols (Figure 1D, and Figure S2C in Supporting Information S1). In Baum et al., a methanol fixation was employed, which may retain only a specific population or may tend to modify the localization during fixation. TgFRM2 showed the same subcellular localization as TgFRM1 (Figure 1E), however, when extracellular parasites were treated with aerolysin, the two formins behaved differently. The pore-forming toxin binds to glycosylphosphatidylinositol (GPIs) anchored proteins and induces an osmotic swelling that selectively detaches the plasma membrane (PM) from the inner membrane complex (IMC) [19]. Upon aerolysin treatment, TgFRM1 remained associated with the PM, whereas TgFRM2 stayed preferentially connected to the IMC (Figure 1F). The nature of the association of TgFRM1 and TgFRM2 with the PM and IMC, respectively, was examined by fractionation at high pH. Complete solubilisation of both formins indicated that electrostatic interactions, either with membrane proteins or with the polar heads of lipids, are responsible for their membrane association (Figure 1G).

The presence of TgFRM1 and TgFRM2 at the pellicle is compatible with a contribution of both formins to actin polymerization during invasion. In the malaria parasite, PfFRM2 was detected in trophozoites and its presence in late schizonts could not be established [12], [20]. To assess the expression of *PbFRM1* and *PbFRM2* (*Plasmodium berghei* formins 1 and 2) throughout the erythrocytic stages a myc epitope tag was inserted at their C-termini of both formins via a knock-in strategy based on single crossover recombination (Figure S3A in Supporting Information S1). The modified locus of the transgenic parasites was confirmed by genomic PCR (Figure S3B and S3C in Supporting Information S1). Western blot analysis revealed that both formins are present in schizonts, leaving open the possibly that both formins play a role in invasion. However, the low level of expression together with the high background of all myc antibodies tested hampered the assessment of their localization by IFA (Figure S3B and S3C in Supporting Information S1).

### TgFRM1 contributes to gliding motility and invasion

**Figure 2:**
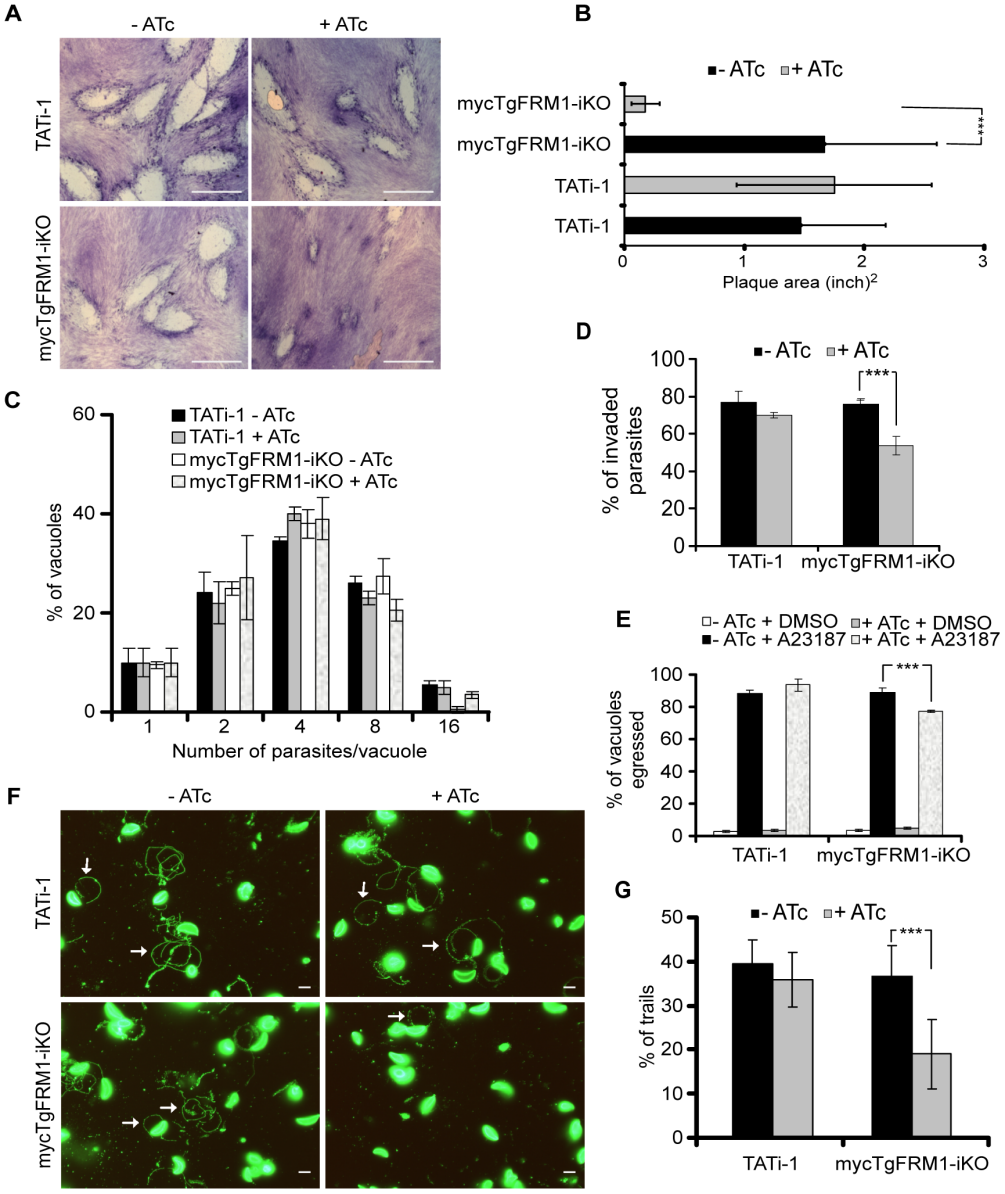
Phenotypic consequences of mycTgFRM1i depletion in mycTgFRM1-iKO. (A) Plaque assay performed on HFF monolayer infected with TATi-1 or mycTgFRM1-iKO parasites. After 6 days ± ATc, the HHF were stained with Giemsa. The scale bar represents 1 mm. (B) The area of 30 plaques formed by the individual strains ± ATc were measured by using the ImageJ program. Values are means ± SD. Statistical significance was evaluated using the unpaired t test. ***P<0.0001. (C) Intracellular growth of TATi-1 and mycTgFRM1-iKO cultivated in presence or absence of ATc for 96 hours and allowed to invade new HFF cells. Numbers of parasites per vacuole (X axis) were counted 24 hours after inoculation. The percentages of vacuoles containing varying numbers of parasites are represented on the Y-axis. Values are means ± SD for three independent experiments. (D) Invasion assay performed on mycTgFRM1-iKO pre-treated ± ATc. IFA was performed with anti-SAG1 prior to permeabilization and with anti-GAP45 after permeabilization. The % of invaded (red) parasites is represented on the graph. Values are means ± SD for three independent experiments. Statistical significance was evaluated using the unpaired t test. ***P = 0.0003. (E) A23187 induced egress assay mycTgFRM1-iKO pre-treated ± ATc. Values are means ± SD for three independent experiments. Statistical significance was evaluated using the unpaired t test. ***P = 0.0003. (F) Gliding assay performed on mycTgFRM1-iKO pre-treated ± ATc. White arrows show trails from both circular and helical gliding stained with anti-SAG1 antibodies. Scale bars represent 2 µm. (G) Parasites were monitored for trail deposition in gliding assay using SAG1 antibodies. Values are means ± SD for three independent experiments. Results are displaced as % of total parasites. The trails depositions from 600 parasites were counted. Statistical significance was evaluated using the unpaired t test. ***P = 0.0003.

The promoter exchange at the *TgFRM1* locus leads to a partial depletion in TgFRM1 upon ATc treatment. The phenotypic consequences were first investigated by plaque assays. The lytic cycle of the parasite is a multi-step process that involves invasion, several rounds of replication and egress. The plaque assay corresponds to plaques of lysis formed in a monolayer of human forskin fibroblasts (HFF) that recapitulates multiple lytic cycles over several days. When mycTgFRM1-iKO parasites were depleted of mycTgFRM1i after six days of ATc treatment, the plaques formed were significantly reduced in size, compared with the untreated mutant and TATi-1 strain (Figure 2A and B). Further analysis established that depletion in TgFRM1 was not affecting parasite replication (Figure 2C) but caused a defect in invasion (Figure 2D; Table 1). The importance of TgFRM1 for parasite egress was investigated upon addition of the calcium ionophore A23187 and only a modest impairment of 14% in induced egress was recorded (Figure 2E and Table 1). To monitor if parasites depleted in TgFRM1 could still accomplish the three forms of gliding movement (helical gliding, circular gliding and twirling), trails deposited by moving parasites on coated Poly-L-lysine cover slips were scored by IFA. The mycTgFRM1-iKO strain showed a significant reduction in trail formation after ATc treatment (Figure 2F, 2G and Table 1). Taken together these results indicated a role for TgFRM1 in invasion, although the phenotype reported here is very modest compared to other invasion factors such as TgMyoA and TgMIC2 investigated before with the Tet-system [4], [21]. Clearly, this system is not ideally suited to study the function of weakly expressed genes. The partial phenotype observed in the conditional knockdown of *TgFRM1* is likely due to the residual amount of mycTgFRM1 (13%) produced in the presence of ATc that corresponds to almost 50% of endogenous levels of TgFRM1. At this stage, it is not possible to exclude that TgFRM1 and TgFRM2 are functionally redundant.

**Table 1:** Phenotypic consequences observed in this study.

Phenotype→	ATc	Intracellular growth	Egress	Gliding	Invasion
Strain↓					
TATi-1	−	Normal	88% ±2	42% ±6	77% ±6
TATi-1	+	Normal	93% ±4	38% ±70	71% ±2
mycTgFRM1-iKO	−	Normal	89% ±3	39% ±7	76% ±3
mycTgFRM1-iKO	+	Normal	77% ±1	20% ±8	53% ±5

Strain	Shield	Intracellular growth	Egress	Gliding	Invasion
RH-hxgprtKO	−	Normal	83% ±3	33% ±5	89% ±3
RH-hxgprtKO	+	Normal	75% ±5	33% ±13	90% ±17
DD-F1-IR/AA	−	Normal	80% ±3	31% ±5	96% ±7
DD-F1-IR/AA	+	Normal	40% ±2	16% ±9	48% ±6
DD-F2-R/A	−	Normal	82% ±4	37% ±10	98% ±16
DD-F2-R/A	+	Normal	34% ±4	14% ±2	49% ±8
DD-F1-IR/AADD-F2-R/A	−	Normal	92% ±2	36% ±8	97% ±11
DD-F1-IR/AADD-F2-R/A	+	Normal	22% ±2	10% ±3	31% ±5
DD-F1	−	Normal	nd.	nd.	nd.
DD-F1	+	Affected	nd.	nd.	nd.
DD-F2	−	Normal	nd.	nd.	nd.
DD-F2	+	Affected	nd.	nd.	nd.
DD-F1-ΔH	−	nd.	nd.	nd.	nd.
DD-F1-ΔH	+	nd.	nd.	nd.	nd.
DD-F2-ΔH	−	nd.	nd.	nd.	nd.
DD-F2-ΔH	+	nd.	nd.	nd.	nd.

Values listed in this table are summarizing the results presented in figures 2 and 5. nd: not done.

### Expression and biochemical analysis of TgFRM1 and TgFRM2 FH2 domains

Dimerization via the FH2 domain is essential for the processive function of formins, with one subunit attached on the barbed end of an actin filament while the other adopts an open configuration to recruit the incoming actin subunit [22]. To examine if the two *T. gondii* formins can nucleate actin filaments, the boundaries of the FH2 domains were delineated (Figure S1B in Supporting Information S1). Recombinant FH2 domains were produced and purified from *E. coli* (Figures S4A, S4C, S5A, and S5B in Supporting Information S1). The FH2 of TgFRM1 (His-F1L) and TgFRM2 (His-F2) were analyzed by gel filtration on Superose 6 10/300 GL. F1L (amino acid positions 4582-5051) corresponds to a N-terminal 48 amino acid extension of the FH2 domain, which is enriched in proline residues and might constitute a divergent FH1 domain (Figure 1A). F1L (56 kDa) and F2 (100 kDa) fractionated as both monomers and dimers (Figure S5C and S5D in Supporting Information S1).

A Ni-NTA-Sepharose bead pull-down assay demonstrated that the FH2 domains bind to TgACT1 when incubated with parasite lysates (Figures S4A, S4C, S5E, and S5F in Supporting Information S1). Although both His-F1L and His-F2 contained a proline rich region, no interaction with myc-TgPRF was observed (Figure S5E and S5F in Supporting Information S1). These results were confirmed *in vivo* by immunoprecipitation (IP) of TgFRM1 and TgFRM2 under native conditions. While a significant amount of TgACT1 was co-IPed with the two formins (Figure S5G in Supporting Information S1), no TgPRF was precipitated (data not shown). Finally, beads coated with His-F1L or His-F2 failed to initiate processive actin assembly in the presence of both bovine and Toxoplasma profilin proteins, in contrast to the behaviour observed with mDia1-FH1-FH2-coated beads [23]. However, the results observed regarding the processive actin assembly in the presence of profilin do not exclude the positive effect of Toxoplasma profilin on actin elongation by Toxoplasma formins 1 and 2, and a new ranges of conditions need to be tested in the future to clarify this point.

### TgFRM1 and TgFRM2 FH2 domains exhibit distinct biochemical properties as actin nucleators

**Figure 3:**
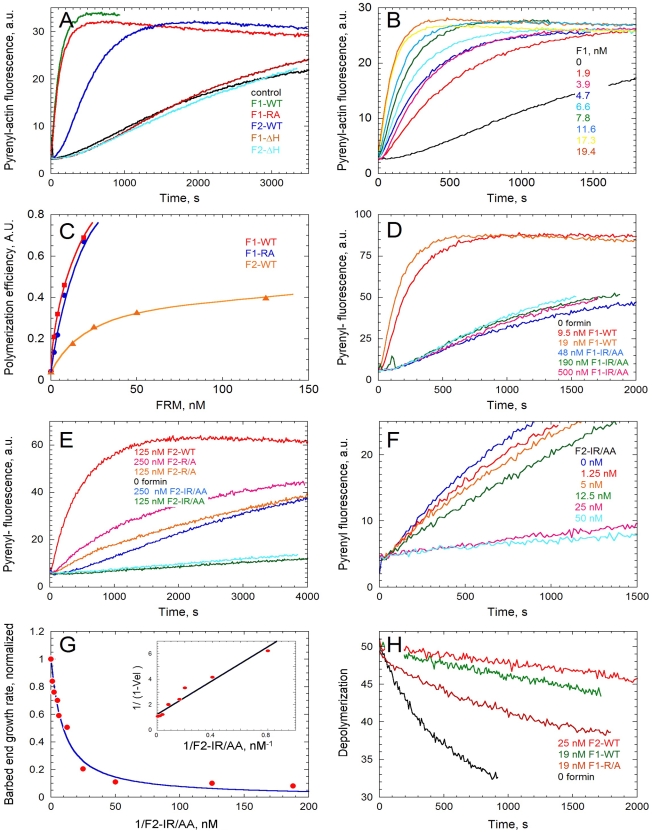
*In vitro* activities of the FH2 domains of TgFRM1 and TgFRM2 in actin assembly. (A) Spontaneous assembly time course of actin at 2.5 µM in the absence (control) and in the presence of 19 nM WT or mutated forms of F1 and F2 as indicated. (B) F1 concentration dependence of the nucleating activity. Actin was polymerized at 2.5 µM in the presence of F1-WT as indicated. (C) Compared nucleating efficiencies of F1, F1-R/A, F2, at different concentrations of formins. (D) The IR/AA double mutation abolishes the nucleating activity of F1. Actin was polymerized in the absence and in the presence of F1-WT or F1-IR/AA as indicated. (E) Effect of R/A and IR/AA mutations on the nucleating activity of F2. Actin was polymerized in the absence and in the presence of F2-WT, F2-R/A or F2-IR/AA as indicated. (F) F2-IR/AA blocks barbed end growth with high affinity. Barbed end growth of actin (2.5 µM) was initiated by spectrin-actin seeds in the absence and in the presence of F2-IR/AA at the indicated concentrations. (G) The initial rate of barbed end growth (from panel F and additional data) was plotted versus the concentration of F2-IR/AA. Inset: double reciprocal plot of the data, indicating that F2-IR/AA blocks barbed ends with a Kd of 6.5 nM. (H) Effect of F1 and F2 on depolymerization of filaments at barbed ends. Depolymerization of filaments was measured by diluting 40-fold a 2.5 µM F-actin solution (70% Pyrenyl-labeled) in F buffer in the absence and presence of F1-WT, F2-WT and F1-R/A as indicated.

The effect of F1 and F2 on actin assembly was tested in spontaneous actin polymerization assays. A qualitative estimation of the nucleating efficiency is derived from the formin concentration dependence of the initial rate of polymerization. F1 strongly stimulated actin polymerization at nanomolar concentrations, while higher concentrations of F2 were required to nucleate actin assembly (Figure 3A, B and C). However, the exact number of nuclei generated by formins cannot be determined from these assays, which do not discriminate between effects on filament nucleation and elongation rate constants. These results generated with rabbit muscle actin, are in agreement with the activities reported for PfFRM1 and PfFRM2 with chicken muscle actin [12].

Crystal structure analysis of the Bni1p FH2 in complex with actin has revealed that an FH2 dimer bridges three consecutive actin subunits arranged along a pseudo-filament, with each FH2 arm contacting two actin subunits [24]. In the pseudo-filament the barbed end is blocked by the FH2 arm, and a conformational change from this “closed” to an “open” configuration had to be postulated to accommodate processive filament growth [24]. The actin-FH2 contacts are made by two highly conserved patches on the surface of the hemidimer. These two sites correspond to Ile1431 and Lys1601 and mutation of either of these residues completely abolishes the actin assembly activity of Bni1 FH2 [24], [25]. The corresponding Lys1601 (residue R4867 in TgFRM1 and R3709 in TgFRM2) were mutated and the resulting FH2 mutants His-F1-R/A and His-F2-R/A were produced and analyzed in actin assembly assays (Figure S1B in Supporting Information S1). Whereas His-F2-R/A lost at least 90% of its nucleating activity, His-F1-R/A still retained a nucleating activity comparable to that of the wild type protein. A second mutation corresponding to Ile1431 (residue I4713 in TgFRM1) was introduced to create the His-F1-IR/AA double mutant, which showed no nucleating activity up to 500 nM (Figure 3C and D). Unexpectedly, the His-F2-IR/AA double mutant (residue I3511 in TgFRM2) displayed a strong barbed end capping activity in filament barbed end growth assays using spectrin-actin seeds (Figure 3E). A value of 6 nM was derived for the equilibrium dissociation constant of the complex of His-F2-IR/AA with barbed ends (Figure 3F and G). This latter result suggests that the double mutation prevents the postulated conformational change of the FH2-barbed end complex that allows processive elongation.

Interaction of these FH2 mutants with barbed ends of filaments was further addressed by monitoring their effect on the initial rate of dilution-induced depolymerization of filaments (Figure 3H). His-F1 totally blocked barbed end depolymerization while His-F2 inhibited the rate of depolymerization by 75% at saturation. Both proteins bound barbed ends with high affinity. His-F1-R/A blocked filament depolymerization from barbed ends as efficiently as His-F1. His-F1-IR/AA did not affect barbed end depolymerization. The R/A mutation did not change the inhibition of depolymerization of the F2, while the double mutation IR/AA abolished the blockage of the barbed end depolymerization harboured by F2 (Figure S5H in Supporting Information S1).

In conclusion, the FH2 domains of TgFRM1 and TgFRM2 display the barbed end binding property common to all formins from other species, but each formin exhibits specific activities at barbed ends. F1 is a more efficient nucleator of actin filaments than F2, but it totally inhibits barbed end depolymerization, which demonstrates its tight binding to the ADP-F-actin terminal subunits that are become exposed during depolymerization. In contrast, F2 only partially inhibits barbed end depolymerization, similar to other formins like mDia1 or Bni1 [23], [26].

### Validation of mutated FH2 domains as dominant negative mutants

**Figure 4:**
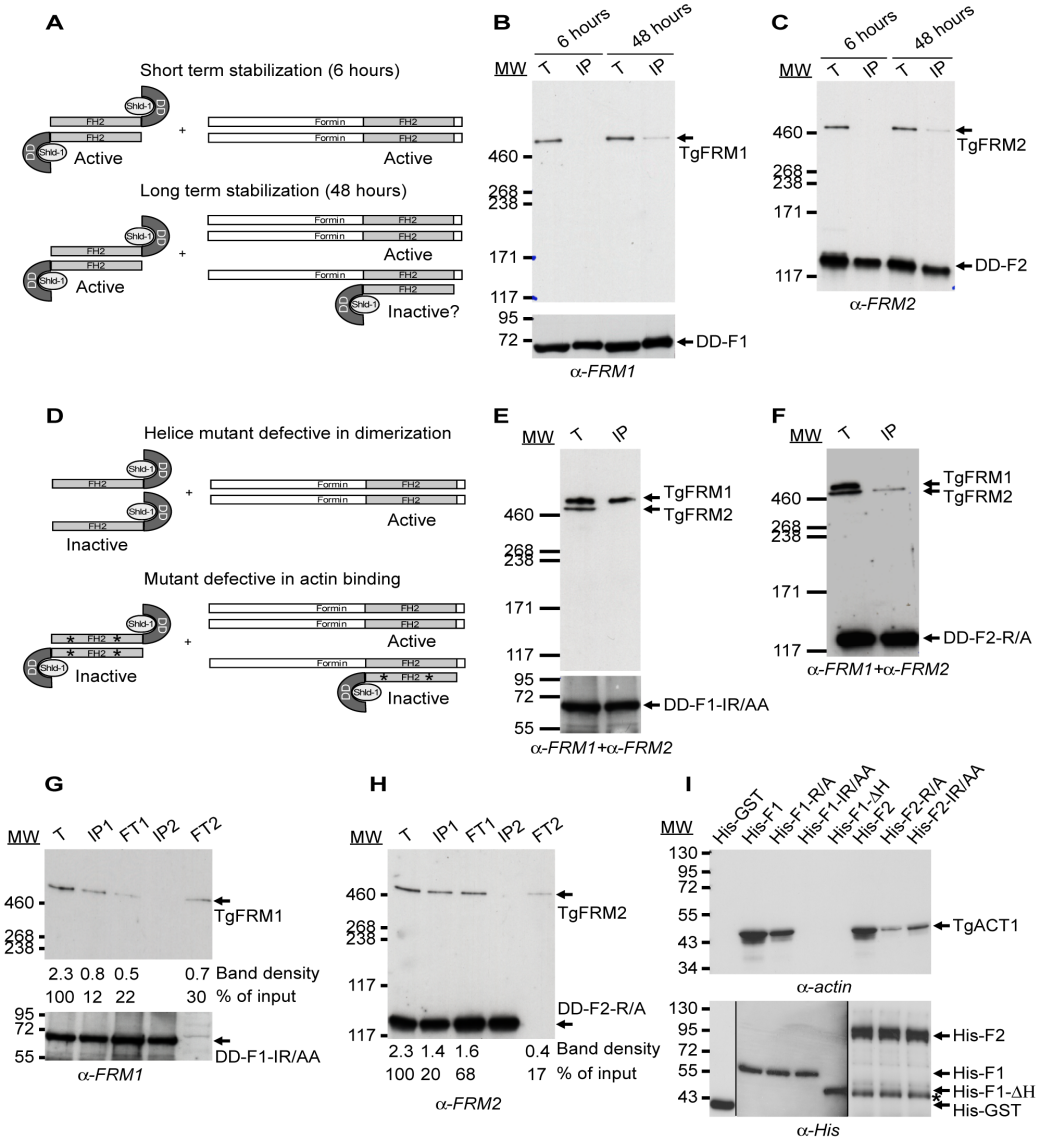
Expression of FH2 domains and their interaction with both endogenous formins and TgACT1 proteins. (A) Schematic representation of the FH2 homodimers fused to DD and FH2/FRM heterodimers upon short and long-term stabilization with Shld-1. (B–C) FH2/FRM heterodimers are formed 48 hrs post stabilization of the FH2 domain. Transgenic parasites were grown in presence of Shld-1 for 6 or 48 hrs prior to harvesting of the parasites and IP with anti-myc antibodies. TgFRM1 (B) and TgFRM2 (C) were monitored by western blot using anti-FRM1 and anti-FRM2 antibodies. Parasite total lysates (T), immunoprecipitated proteins eluted from beads (IP). (D) Schematic representation of FH2-ΔH mutants fused to DD and lacking the two helices responsible for dimerization. Point's mutations represented by asterisks caused a defect in actin binding without affecting dimerization. (E–F) DD-F1-IR/AA and DD-F2-R/A formed selective heterodimers with their corresponding formin. Transgenic parasites were grown in presence of Shld-1 for 48 hrs prior to harvesting of the parasites and IP with anti-myc antibodies. Membranes were probed simultaneously with anti-FRM1 and anti-FRM2 antibodies. (G–H) Depletion of the total lysates from F1-IR/AA/FRM1, F1-IR/AA/F1-IR/AA, F2-R/A/FRM2, and F2-R/A/F2-R/A by two sequential immunoprecipitations with anti-myc antibodies. Total lysates (T) or immunoprecipitated proteins eluted from beads (IP1 and IP2) or lysates after immunoprecipitation (flow through 1 (FT1) and FT2) were analysed by Western blots. Membranes were probed with either anti-FRM1 (G) or anti-FRM2 (H) antibodies. The integrated densities of the bands measured using the ImageJ program, and the values expressed in percentage of the total input are provided after normalization for equal loading. (I) Nickel affinity pull-down assay, which measured the ability of FH2 domains fused to His to bind to TgACT1. The amount of TgACT1 and FH2 domains were determined by Western blot analysis using anti-actin and anti-His antibodies. The asterisk represents F2 domains degradation.

Although a role in invasion could be established, the partial depletion in TgFRM1 hampered a proper assessment of the importance of the gene. Moreover, the lack of success in generating a conditional knockout for *TgFRM2* impeded a functional assessment of its contribution. To overcome these technical limitations, we developed an approach based upon the conditional expression of a dominant negative mutant that would be suited to study selectively the function of multiple formins in the same cell. We reasoned that the expression of a FH2 domain should lead to the formation of a defective heterodimer (FH2-FRM). Given that the FH2 WT domain forms homodimers that possess an unregulated capacity to polymerize actin, it was crucial to prevent such activity by introducing mutations in the actin-binding site (F1-IR/AA and F2-R/A). In this scenario both homodimers and heterodimers are predicted to be inactive (Figure 4A). As negative controls, truncated forms of FH2 (F1-ΔH, and F2-ΔH) that lack the two helices required for dimerization were generated (Figures S1B and S4 in Supporting Information S1) [12], [27].

To circumvent the anticipated deleterious effect caused by the expression of these mutants on parasite survival, both the WT and mutated FH2 domains were fused to the destabilization domain (DD) of FKBP. This small domain is known to confer instability to proteins in the absence of the folding inducer shield molecule, Shld-1 [28], [29]. Transgenic parasites expressing WT, deletion mutants unable to dimerize as well as site-specific mutants unable to bind to actin, were generated for both formins in the type I RH laboratory strain of *T. gondii* (Figure S4B and S4D in Supporting Information S1; Table S2 in Supporting Information S1). The tight control of DD-FH2 fusions by Shld-1 was assessed by western blot and IFA (Figure S6A and S6B in Supporting Information S1).

Since formins generally form extremely stable dimers (except maybe mDia2, [30]), the expression of DD-FH2 is not anticipated to form hybrids from preformed endogenous dimers but only to associate with newly synthesized proteins. Given the likely slow turnover of such large proteins, the formation of DD-F1/FRM1 and DD-F2/FRM2 heterodimers was monitored 6 and 48 hours following Shld-1 treatment. IP of DD-F1 and DD-F2 was performed under native conditions and revealed that no heterodimers were formed after 6 hours of Shld-1 treatment. In contrast, significant amounts of heterodimers were detectable at 48 hours (Figure 4B, and 4C; Figure S6C and S6D in Supporting Information S1). As anticipated, no heterodimers were formed with DD-F1-ΔH and DD-F2-ΔH (Figure 4D; Figure S6C and S6D in Supporting Information S1). These results highlight the functional conservation of the elements required for the self-association of formins across species [9]. In contrast, DD-F1-IR/AA, and DD-F2-R/A mutants impaired in actin binding and nucleating activity were associated with their corresponding formins with the same efficiency as DD-F1 and DD-F2 respectively (Figure 4D, 4G, and 4H; Figure S6C and S6D in Supporting Information S1). Importantly, the dimerization is specific for each formin as DD-F1-IR/AA, and DD-F2-R/A were exclusively associated with their corresponding formins, as shown by Western blot analysis of the co-IPs in presence of both anti-FRM1 and anti-FRM2 antibodies (Figure 4E, and 4F). Despite the high level of DD-F1-IR/AA and DD-F2-R/A expression, the coIP experiments revealed that endogenous TgFRM1 and TgFRM2 were not completely sequestered as heterodimers. Even after the second coIPs, when DD-F1-IR/AA and DD-F2-R/A were completely depleted in the second flow through (FT2), both formins were still present TgFRM1 (ca. 30%) and TgFRM2 (ca. 17%) compared to the inputs (Figure 4G, and 4H; lanes corresponding to FT2). Prior to the assessment of their dominant negative effects in *T. gondii*, the FH2 constructs, expressed as His-fusion and purified from *E. coli*, were assessed for binding to TgACT1 by pull down assays with parasite lysates. While His-F1 and His-F2 bound efficiently to TgACT1, no binding was detected with His-F1-ΔH, establishing that dimerization of the FH2 domains is necessary for actin association (Figure 4I upper panel). His-F2-ΔH is very unstable after its purification from bacteria, and was therefore not included in this analysis. In agreement with the polymerization assays, His-F1-IR/AA did not bind to actin, whereas His-F1-R/A showed residual binding (Figure 4I upper panel). His-F2-R/A was considerably impaired in binding to TgACT1, whereas His-F2-IR/AA showed an increased ability to bind to TgACT1 consistent with the barbed end capper activity detected in polymerization assays (Figure 4I upper panel). To consolidate these results, we verified that all recombinant FH2 proteins bound quantitatively to the nickel column (Figure 4I lower panel).

### TgFRM1 and TgFRM2 contribute to parasite motility and host cell invasion

**Figure 5:**
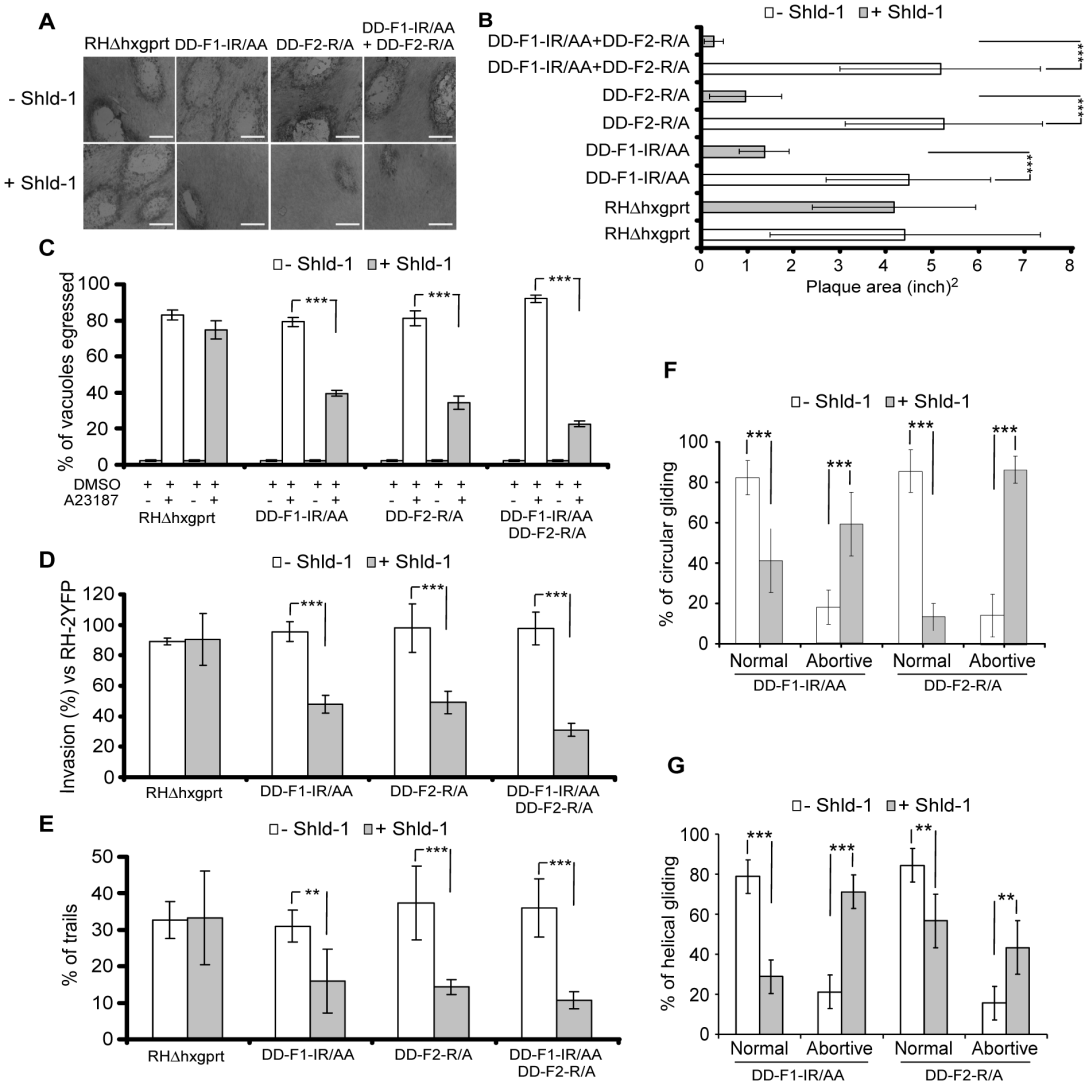
Phenotypic analysis of parasites expressing FH2 domains. (A) Plaque assays on transgenic parasites upon stabilization of FH2 after 6 days. The scale bar represents 1 mm. (B) The area of 30 plaques formed by the individual strains ± Shld-1 were measured by using the ImageJ program. Values are means ± SD. Statistical significance was evaluated using the unpaired t test. ***P<0.0001. (C) Egress assay on transgenic parasites upon stabilization of FH2 for 63 hrs. Egress was triggered by 3 µM of A23187. The extent of vacuole lysis and parasite spreading were scored by visual examination on IFA. Values are means ± SD for three independent experiments. ***P<0.0001. (D) Invasion assay on transgenic parasites upon stabilization of FH2 for 63 hours. Parasites were allowed to invade new HFF cells for 24 hours. Values are means ± SD for three independent experiments. ***P = 0.0007, ***P<0.0001, ***P<0.0001 respectively. (E) Parasites were treated as in (C) and monitored for trail deposition in gliding assay using SAG1 antibodies. No difference in trail deposition was seen for RHΔhxgprt strain upon Shld-1 treatment. Values (n = 600 parasites) are means ± SD for three independent experiments. **P = 0.0046, ***P = 0.0008, ***P = 0.0002 respectively. (F) Phenotypic analysis of circular gliding movement in live gliding parasites; error bars represent standard deviation. Values (n = 30 parasites) are means ± SD for three independent experiments. ***P = 0.0007, ***P = 0.0007, ***P<0.0001, ***P<0.0001 respectively. (G) Phenotypic analysis of helical gliding movement in live gliding parasites; error bars represent standard deviation. All data of this figure represent mean values of three experiments. Values (n = 30 parasites) are means ± SD for three independent experiments. ***P<0.0001, ***P<0.0001, **P = 0.0025, **P = 0.0025 respectively.

The heterodimers formed between endogenous FRM1 and DD-F1-IR/AA or FRM2 and DD-F2-R/A are predicted to be non-functional and hence to mimic the knockdown of the corresponding gene. In contrast, wild type F1 and F2 form active homodimers that could lead to some pleiotropic effects as a consequence of uncontrolled actin polymerization. Indeed, stabilization of DD-F1 and DD-F2 were severely impaired in plaque formation due to a strong growth defect (Figure S7A, S7B, and S7C in Supporting Information S1). Stabilization of DD-F1-ΔH and DD-F2-ΔH showed no defect thus ruling out any deleterious effect resulting from the stabilization of DD-FH2 without the ability to form a functional dimer (Figure S7A in Supporting Information S1). To monitor TgFRM2 function, we excluded DD-F2-IR/AA which exhibits a yet unexplained capping activity (Figure 3E, F and G), and used instead DD-F2-R/A, which is impaired in nucleating activity (Figures 3E, and 4I). Generation of parasite lines expressing both DD-F1-IR/AA and DD-F2-R/A allowed assessment of TgFRM1 and TgFRM2 function simultaneously. Stabilization of DD-F1-IR/AA and DD-F2-R/A highlighted the importance of both formins by plaque assays (Figure 5A, and B). Importantly, when compared to the same strains untreated with Shld-1, these mutants did not alter intracellular growth, excluding unspecific toxic effect (Figure S7D in Supporting Information S1). In contrast, less than 50% of parasites expressing DD-F1-IR/AA and 40% of those expressing DD-F2-R/A were able to egress while only 23% of egress was observed for parasites expressing both FH2 mutants (Figure 5C; Table 1). Invasion efficiency of each mutant was normalized to the invasion efficiency of a YFP strain (taken as 100%). With regards to egress, a partial defect was observed with F1-IR/AA (50%) or F2-R/A (50%), and an enhanced defect of 68% upon co-expression (Figure 5D; Table 1). Similarly, parasites expressing DD-F1-IR/AA; DD-F2-R/A and DD-F1-IR/AA+DD-F2-R/A showed a 49%, 62%, and 72% defect in trail formation, respectively (Figure 5E; Table 1).

Gliding defects were examined in more depth by live video microscopy. In presence of Shld-1, DD-F1-IR/AA and DD-F2-R/A parasites exhibited normal twirling motion but were defective in circular and helical motion. Only 41% of the parasites expressing DD-F1-IR/AA and 13% expressing DD-F2-R/A were able to accomplish a complete multi-circular movement lasting up to one minute (Figure 5F; Video S1). In contrast, up to 80% of non-treated parasites exhibit normal circular gliding as previously described [31] (Figure 5F; Video S1). While interference with TgFRM2 function showed a more pronounced impairment on circular gliding (Figure 5F; Videos S2 and S3), the inhibition of TgFRM1 function caused a preferential defect in helical gliding with 71% of the parasites expressing DD-F1-IR/AA being affected compared to 43% of those expressing DD-F2-R/A (Figure 5G; compare Video S4 with Video S5).

## Discussion

Gliding motility is a dynamic event involving temporally and spatially controlled actin polymerization. Typically, formins assisted by profilin, bind to an FH1 domain to facilitate a rapid processive assembly of actin filaments [14], [23], [32]. Despite the importance of TgPRF in parasite motility and invasion [13], none of the parasite formins carry a canonical FH1 domain. Since the region right upstream of FH2 domains of both formins is rich in short stretches of proline residues their potential to act as binding site for profilin was investigated but i*n vitro* pull-down experiments and *in vivo* coIPs failed to show interaction. New subclasses of formins apparently lack FH1, suggesting that an FH1-independent pathway may mediate actin assembly [9], [33]. The crystal structure of the Plasmodium PRF in complex with an octa-proline peptide was solved and implicated the N-terminal tyrosine residue (Tyr5) in tethering to the poly-proline [34]. In our hands, all apicomplexan PRFs showed either very low or no affinity for poly-proline (Plattner F., unpublished). It is plausible that the apicomplexan PRFs bind to a divergent unrecognizable domain on formins and further work is required to unravel how TgPRF contributes to actin filament formation.

TgFRM1 and TgFRM2 bind to TgACT1 and share biochemical characteristics with their counterparts in Plasmodium [12]. Both formins are underneath the PM and aerolysin treatment revealed their differential affinity to membranes within the narrow space separating the IMC from the PM. Like TgMyoA, both formins homogenously distribute at the periphery of invading parasites, compatible with the notion that they may nucleate actin at any time and at any point of contact between the parasite and its substrate.

Conditional knockout of TgFRM1 established its role in motility and invasion although the effects were modest and impact on egress was minor. Despite multiple attempts, generation of a conditional knockout for *TgFRM2* failed. In the course of this study, parasite lines were generated with triple Ty-1 tags inserted at the C-terminus of each formin by single crossing over in the ku-80-ko strain. RT-PCR analysis confirmed in frame integration of the tags but no signal was detectable by IFA or Western blot in these transgenic parasites (data not shown). These results indicate that the endogenous levels of both formins are extremely low and hence explain the weak phenotype observed upon mycFRM1i depletion. In the same context, the lack of success in replacing the endogenous TgFRM2 promoter with an inducible promoter might be due to a deleterious effect of mycFRM2i expression if the level is too high.

The function of TgFRM2 and possible redundancy with TgFRM1 was assessed by the expression of FH2 mutants to poison individually or simultaneously the two endogenous formins. This strategy also showed some limitations since the co-IP experiments revealed that 30% of FRM1 and 17% of FRM2 were not sequestered in defective heterodimers. This suggests that the affinity and or the stability of the homodimers (FRM-FRM and FH2-FH2) are higher than the heterodimer (FRM-FH2).

The FH2 WT, F1 and F2 are potent actin nucleators and their overexpression had a severe impact on parasite replication that was not dependent on TgFRM1 and TgFRM2. Points mutations were introduced in the FH2 domains to disrupt actin nucleation and hence eliminate this non-specific effect. As with Bni1p [25], a single point mutation in TgFRM2 (F2-R/A) was sufficient to abrogate its activity whereas a double mutation (F1-IR/AA) was needed to abolish actin nucleation of TgFRM1. The IR/AA double mutation conferred to F2 an unexpected barbed end capping activity. This mutation may impair the flexibility of the FH2 domain and prevent the switch from the closed to the open configuration during elongation. To understand this phenomenon, the resolution of the FH2 domain structure of TgFRM2 in presence of actin would be necessary.

The different effects of the R/A, and IR/AA mutations on the activities of TgFRM1 and TgFRM2 further testify that these two formins have different modes of interaction with actin. The fact that mutations affect differently barbed end growth and depolymerization processes, in which ATP/ADP-Pi-actin and ADP-actin are respectively exposed at barbed ends, suggests that these mutations may affect their interactions with ATP-actin and ADP-actin differently. Similar differences have already been observed with twinfilin, a capping protein that binds preferentially to ADP-bound barbed ends [35]. The R/A mutation does not affect any of the activities of TgFRM1. In contrast, the R/A mutation of TgFRM2 may weaken its interaction with ATP-actin but not with ADP-actin. The IR/AA double mutation abolishes all activities of TgFRM1. The same double mutation transforms TgFRM2 into a strong barbed end capper in nucleation and barbed end growth, while leaving the barbed end depolymerization unaffected, which suggests that the double mutation reinforces binding of FRM2 to the ATP-terminal subunits in its “closed” configuration and abolishes its binding to ADP-terminal subunits.

Stabilization of DD-F1-IR/AA and DD-F2-R/A did not affect intracellular growth and revealed that both TgFRM1 and TgFRM2 play a role in gliding, invasion and egress. All phenotypes were aggravated when both dominant mutants were expressed in the same parasite (Table 1). These results give a strong indication that the two formins act in concert. However, since the stabilization of each FH2 mutants failed to sequester all the formins, invasion only dropped to 50% and in consequence it is not possible to completely rule out some level of functional redundancy between the two formins. Nevertheless the results demonstrate that both TgFRM1 and TgFRM2 contribute additively to the three vital aspects of the glideosome function namely gliding motility, host cell invasion and egress from the infected cells.

The refined analysis of the gliding motility phenotypes by video microscopy revealed that interfering with TgFRM1 and TgFRM2 preferentially affected helical and circular gliding, respectively, illustrating distinct contributions of the two formins in gliding. This study revealed that TgFRM1 is preferentially positioned at the PM, where fast nucleation occurs in close proximity to the complex formed between actin filaments and the aldolase-MIC2 tail complex. The filaments likely elongate over only a short distance with TgFRM2 potentially serving to stabilize and control the size of the filament close to the IMC.

Given the importance of these formins for parasite infection, it will be imperative to elucidate their mode of regulation and interaction with profilin as these unique features might become relevant therapeutic targets.

## Materials and Methods

### Parasites culture and cloning of genes

*T. gondii* tachyzoites (RH *hxgprt*-ko, or TATi-1) were grown in human foreskin fibroblasts (HFF). Selections of transgenic parasites were performed with mycophenolic acid (MPA) and xanthine for HXGPRT selection [36]; chloramphenicol for CAT selection [37]; anhydrotetracycline (ATc) for the inducible system [38]; 1 µM Shld-1 for DD-fusion stabilization [29]; pyrimethamine for DHFR-TS selection [39].

### Toxoplasma and *E. coli* vectors

Primers used in this study are listed in the Table S1 in Supporting Information S1. The ptetO7Sag4mycNtTgFRM1-KO: A genomic fragment of 1513 pbs corresponding to the N-terminal coding sequence of *TgFRM1* gene was amplified by PCR subcloned into *Nsi*I and B*am*HI sites of ptetO7Sag4mycGFP. The 5′ flanking region of *TgFRM1* promoter was amplified by genomic PCR and cloned into the *Apa*I in pTub5CAT. The ptetO7Sag4mycNtTgFRM1 cassette was subcloned into the *Sac*I site of pTub5CAT.

The ptetO7Sag4mycNtTgFRM2-KO: A genomic fragment of 2202 pbs corresponding to the N-terminal coding sequence of *TgFRM2* gene was amplified by PCR subcloned into *Nsi*I and B*am*HI sites of ptetO7Sag4mycGFP. The 5′ flanking region of *TgFRM2* promoter (2633 pbs) was amplified by genomic PCR and cloned into the *Apa*I in pTub5CAT. The ptetO7Sag4mycNtTgFRM2 cassette was subcloned into the *Sac*I site of pTub5CAT.

The series of pTub8DDFKBPmycFH2 vectors were obtained by cloning of the FH2 cDNAs into *Nsi*I and *Pac*I in pTub8DDFKBPmyc vector.

To mutate the Isoleucine (I) or the Arginine (R) residues, primers described in the Table S1 in Supporting Information S1 were used in a site-directed mutagenesis reaction using the commercial QuikChange II Site-DirectedMutagenesis Kit (Stratagene) and according to manufacturer's instructions. All mutated constructs were sequenced along the entire open-reading frame (ORF) to confirm the correct sequence.

The bacterial expression was achieved by insertion of wild type, truncated and mutated F1 and F2 between *Nco*I and *Eco*RI in both pETHTB and pETM30 vectors. F1L (amino acids numbers 4582-5051), F1 (amino acids numbers 4630–5051), F1-R/A (amino acids numbers 4630–5051, R4867/A), F1-IR/AA (amino acids numbers 4630–5051, R4867/A and I4713/A), F1-ΔH (amino acids numbers 4684–5051), F2 (amino acids numbers 3317–4043), F2-R/A (amino acids numbers 3317–4043, R3709/A), and F2-IR/AA (amino acids numbers 3317–4043, R3709/A and I3511/A) were cloned into pETHTB vector to generate recombinant proteins fused to a His tag. F1L (amino acids numbers 4582–5051), F1 (amino acids numbers 4630–5051), F1-R/A (amino acids numbers 4630–5051, R4867/A), F1-IR/AA (amino acids numbers 4630–5051, R4867/A and I4713/A), F1-ΔH (amino acids numbers 4684–5051), F2 (amino acids numbers 3317–4043), F2-R/A (amino acids numbers 3317–4043, R3709/A), F2-IR/AA (amino acids numbers 3317–4043, R3709/A and I3511/A), and F2-ΔH (amino acids numbers 3480–4043) were cloned into pETM30 vector to generate recombinant proteins fused to both His and GST tags. The pET3amycHisF1 and pET3amycHisF2 were used to produce the FH2 for immunization.

The knock-in constructs for *P. berghei* pSD141/CtPbFRM1 and pSD141/CtPbFRM2 were generated by genomic PCR amplification of 1800 bps and 1812 bps corresponding to the C-terminal part of the *PbFRM1* and *PbFRM2* genes, respectively. The PCR products lacking the stop codon were cloned between *Kpn*I and *Apa*I of pSD141 vector in fusion with two myc tags [40].

### Generation of transgenic *T. gondii* and *P. berghei* strains

TATi-1 were transformed with 100 µg of 5′flanking tetO7Sag4mycNtTgFRM1-KO vector (linearized with *SfoI*) and subjected to chloramphenicol selection. YFP-negative parasites were recovered using a FACS sorter to collect negative cells. DD-FH2 expressing parasites were obtained in RH*hxgprt*- and selected for MPA resistance. F1-IR/AA expressing parasites were co-transformed with linearized 90 µg pTUB8-DD-myc-F2-R/A and 10 µg p2854-DHFR.

Single crossing over events in *PbFRM1* and *PbFRM2* loci were obtained as described [41], [42]. The *P. berghei* ANKA strain clone 2.34 [43] was injected intraperitoneally into CD1 mice. The parasitized erythrocytes were harvested after *in vitro* maturation. Linearized plasmid DNA was transfected into purified schizonts using Amaxa machine (Biorad company), and pyrimethamine selection was performed [41]. Pools of parasites resistant to pyrimethamine were genotyped and analyzed by Western blot.

### Protein purification and analysis

His tagged proteins were purified on Qiagen Ni-NTA superflow resin (30410) under native conditions [44]. GST tagged proteins were purified on Amersham Glutathione sepharose 4 Fast flow (17-5132-01) in Amersham 10/20 Tricorn column (18-1163-13). GST-TgPRF was cleaved using the Prescission protease (Amersham, 27-0843-01). The purified FH2 domains were eluted at the rate of 0.4 ml/min with PBS-NaCl 0.15 M buffer, with a Superose 6 10/300 GL column using AKTA prime machine (Amersham Pharmacia biotech) to determine their oligomeric state. To detect TgFRM1 and TgFRM2, parasite lysate were fractionated on Tris-Acetate 3–8% precast gels (Invitrogen) using the manufacturer's running buffer and electrophoresis was continued until the 71–117 kDa marker reached the bottom of the gel. To compare the amount of mycTgFRM1 protein in presence or in absence of ATc with the endogenous level of expression of FRM1, a western blot analysis was performed by loading an equal volume from the total protein extracts derived from both TATi-1 and mycTgFRM1i KO strains. The quantification of the bands was processed using ImageJ program.

### Antibodies and indirect immunofluorescence assay (IFA)

His-F1 and His-F2 were used to immunize rabbits (Eurogentec). Anti-catalase (CAT), anti-TgPRF, anti-SAG1, anti-TgGAP45, anti-IMC1, anti-MLC1, anti-ACT, anti myc (9E10) and anti-Ty tag (BB2) were previously described [13], [45], [46]. Anti-RON4 was kindly provided by Dr. Dubremetz. Immunoblots were visualized using a chemiluminescent substrate (Amersham, GE healthcare).

HFF cells infected with parasites were fixed 15 minutes at room temperature (RT) with 4% paraformaldehyde (PFA) in PBS or 4%PFA/0.05% glutaraldehyde (PFA/GA) in PBS depending on the antigen to be labelled. Cells were neutralized 3–5 minutes in 0.1 M glycine/PBS, and then permeabilized with 0.2% Triton/PBS for 20 minutes. Cells were then incubated with primary antibody (diluted in 2%BSA/0.2% triton/PBS) for 1 hour at RT on balance, washed 3 times with 0.2% Triton/PBS and incubated with secondary antibody as above. Cells were washed 3 times, stained for 5 minutes with DAPI (50 µg/ml in PBS) and washed again. Coverslips were mounted with Fluoromount G (Southern Biotech 0100-01) on glass slides [47].

### Nickel affinity pull-downs

Parasites expressing mycPRF were used as source of PRF and F1L, F1, and F2 were fused to GST and shown to polymerize rabbit actin. Freshly egressed parasites (3×10^8^ parasites) were harvested, washed once with buffer G (CaCl_2_ 0.1 mM, Tris 5 mM pH 7.8, ATP 0.2 mM, and DTT 1 mM), and resuspended in the same buffer containing 0.5 mM ATP and protease inhibitors. Successive rounds of freeze/thaw in liquid N_2_ were performed to break the cells. After ultracentrifugation at 30000 rpm, the supernatant was incubated for 2 hours at 4°C with 75 µg of the bait protein (His-GST or His-GST-F1 or His-GST-F1-R/A or His-GST-F1-IR/AA or His-GST-F1-ΔH or His-GST-F2 or His-GST-F2-R/A or His-GST-F2-IR/AA) followed by incubation with 50 µl of Nickel beads (Qiagen) for 1 hour at 4°C. Beads were centrifuged and an aliquot of the supernatant was taken (flow through). Beads were washed 3 times with buffer G (CaCl_2_ 0.1 mM, Tris 5 mM pH 7.8, ATP 0.2 mM, and DTT 1 mM), suspended in protein loading buffer, and analysed by western blot.

### Immunoprecipitation

FRM1-DD-myc-F1 or FRM1-DD-myc-F1-IR/AA and FRM2-DD-myc-F2 or FRM2-DD-myc-F2-R/A heterodimers complexes were immunoprecipitated with monoclonal 9E10 anti-myc antibodies. To achieve this, 3×10^8^ parasites were lysed in PBS/0.2% triton-X100. Incubation for 1 hour at 4°C with an excess of antibodies was followed by incubation with 25 µl of protein A beads for 1 hour at 4°C. Beads were then washed 3 times with washing buffer, suspended in protein loading buffer, and analysed by Western blot using rabbit polyclonal anti-FRM1 and anti-FRM2 antibodies. To quantify how much endogenous formin was sequestered by the corresponding FH2 mutant, two sequential immunoprecipitation experiments were performed. The two immunoprecipitated fractions and the two flow throughs were analysis by Western blot. The quantification of the bands was processed using ImageJ program and nomalized to the % of FRM present in the input (total lysate).

### Fractionation

Parasites were harvested and extracted in the following buffers: PBS or PBS/Na_2_CO_3_ (0.1 M, pH 11.5). Extracts were then centrifuged at 30000× rpm for 1 hour at 4°C. Equivalent amounts of total, supernatant, and pellet were run on Tris-Acetate 3–8% precast gels (Invitrogen) for formins 1 and 2, and on 10% gel for catalase.

### Aerolysin treatment

Cover slips were coated with a solution of Poly-L-Lysine. Prior to use recombinant protoxin was activated for 20 minutes at 37°C in 100 µl of PBS with 2 µl of trypsin diluted at 1 mg/ml into HBS (140 mM NaCl, 2.7 mM KCl, 20 mM Hepes pH 7.4). Freshly harvested parasites were washed with PBS and attached to coverslips coated with Poly-L-Lysine (incubation at 37°C for 10 minutes). The medium was then removed, and parasites were treated with aerolysin at 60 ng/ml for 3 hours at 37°C and then IFA was performed as described.

### Polymerization assays

Actin was purified from rabbit muscle acetone powder and isolated in monomeric form by gel filtration on Superdex-200 in G buffer. Spontaneous assembly of actin was monitored using the enhancement of the fluorescence of 5% pyrenyl-labeled actin in a Safas Xenius spectrofluorimeter. Conditions were: 2.5 µM actin, 5 mM tris-Cl- pH 7.8, 0.2 mM ATP, 1 mM DTT, 0.1 mM CaCl_2_, 0.25 mM EGTA, 1 mM MgCl_2_, 0.1 M KCl. Seeded actin assembly assays were performed similarly using spectrin-actin seeds and 2.5 µM G-actin [48]. Dilution-induced depolymerization assays were performed by diluting 40-fold a solution of 2.5 µM F-actin (50% pyrenyl-labeled) in polymerization buffer containing the desired concentrations of formins. The initial rate of fluorescence decrease was measured [23].

### Plaque assay

Fresh monolayers of HHF on circular coverslips were infected with parasites in the presence or absence of 1 µg/ml ATc and 1 µM Shld-1 for 6 days. Fixation, staining and visualization were performed as previously described [13].

### Intracellular growth assay

Parasites were pretreated for 96 hours with or without ATc or 63 hours with or without 1 µM Shld-1, collected promptly after egress and inoculated onto new HHF monolayers. 24 hours later, the culture was fixed with PFA and stained with anti-TgGAP45. The number of parasites per vacuole was counted for more than 100 vacuoles under each condition.

### Invasion assay

Freshly released parasites were inoculated onto new confluent HHF monolayer and allowed to invade for 1 hour before the cells were fixed. IFA was performed as previously described [13]. Comparison of *T. gondii* dominant negative mutant strains for invasion efficiency was done in the presence of the RH-2YFP strain as an internal standard as previously described [49]. Parasites were grown for 63 hours ±1 µM Shld-1.

### Egress assay

After 33 hours of intracellular growth, most vacuoles contain 16–32 parasites. Media was changed and incubated for 8 minutes at 37°C with DMEM containing 0.06% of DMSO or 3 µM of the Ca^2+^ ionophore A23187 (from *Streptomyces chartreusensis,* Calbiochem 100105) as previously described [13].

### Gliding motility assay

Freshly released tachyzoites were collected by centrifugation, resuspended in 100 µl and deposited onto Poly-L-Lysine coated coverslips (1 mg/ml, 2 hrs at RT) in a wet environment for 15 minutes at 37°C previously. Parasites were fixed with PAF/GA and IFA using the anti-SAG1 antibody was performed to visualize the trails.

### Video microscopy of gliding motility

Freshly released parasites were resuspended in Ringer medium, and allowed to glide on glass-bottom dishes (MatTek Corp, Ashland, MA) precoated with poly-L-lysine (1 mg/ml). Video microscopy was conducted using a spinning disk confocal microscope (Ultraview) equipped with Andor Revolution under bright field illumination and in a temperature-controlled stage to maintain 37°C. Images were collected in real time under low-light illumination using an intensified Andor DU-897 E camera with a 60× objective (Nikon Plan Apo NA 1.4 Oil). Videos were recorded at 1.47 frames per second in a total time of 1 minute 8 seconds with a resolution of 0.26 µm/pixel. The video signal was processed using ImageJ program.

### Accession numbers

*Toxoplasma gondii* Formin 1, TgFRM1 (ACY06261); *Toxoplasma gondii* Formin 2, TgFRM2 (ACY06262).

## Supporting Information

Supporting Information S1 Tables S1 and S2; Figures S1 to S7.
(8.93 MB PDF)

Video S1 Live circular gliding video at 5X speed of DD-F2-R/A parasites none treated with Shld-1.
(1.19 MB AVI)

Video S2 Live abortive circular gliding video at 5X speed of DD-F1-IR/AA parasites treated with Shld-1.
(0.89 MB AVI)

Video S3 Live abortive circular gliding video at 5X speed of DD-F2-R/A parasites treated with Shld-1.
(0.82 MB AVI)

Video S4 Live helical gliding video at 5X speed of DD-F1-IR/AA parasites none treated with Shld-1.
(1.51 MB AVI)

Video S5 Live abortive helical gliding video at 5X speed of DD-F1-IR/AA parasites treated with Shld-1.
(1.28 MB AVI)
