# Supplementary material for: Concerted Action of Two Formins in Gliding Motility and Host Cell Invasion by Toxoplasma gondii
Source: PLoS Pathog. 2010 Oct 7;6(10):e1001132. doi: 10.1371/journal.ppat.1001132 (PMC2951370; doi:10.1371/journal.ppat.1001132)
Supplement: Supporting Information S1 — Tables S1 and S2; Figures S1 to S7. (8.93 MB PDF) [file ppat.1001132.s001.pdf]

Table S1: list of primers used in this study.

Number	5'→3' sequence	Restriction site(	Plasmid/function (Forward or reverse)
1	CCGCCTGCAGGCGTCTTGCCCGGCAAGCTGCC	*SbfI*	ptetO7Sag4mycNtTgFRM1 (F)
2	GGATCCTTCAAATCGCTCTTTGCCGGAG	*BamHI*	ptetO7Sag4mycNtTgFRM1 (R)
3	GGGCCCGGCGCCCGAGAAACGGAAAGCTCTC TTCGCC	*ApaI, SfoI*	ptetO7Sag4mycNtTgFRM1-iKO (F)
4	GGGCCCGGCGATCGAAACGGAAGCCCC	*ApaI*	ptetO7Sag4mycNtTgFRM1-iKO (R)
5	ATGCATGAACAAAAACTCATCTCAGAAGAGGATC TGATGGAGAATTTCCGACCCGGTCC	*NsiI*	ptetO7Sag4mycNtTgFRM2 (F)
6	GGATCCTTCCGCTTCTGCCAGTGTCTCG	*BamHI*	ptetO7Sag4mycNtTgFRM2 (R)
7	GGGCCCGTTTAAACCGCAGTATCCTCCAAGTAC CCCC	*ApaI, PmeI*	ptetO7Sag4mycNtTgFRM2-iKO (F)
8	GGGCCCGCGTGGTGAAGCTAAAATGAGAAAGTC	*ApaI*	ptetO7Sag4mycNtTgFRM2-iKO (R)
9	CCTGCAGGCCATGCGGTCCGAAACCATGTCG	*SbfI*	DD-myc-F1 (F)
10	TTAATTAATCACTCACCGACTTTCTTGACTGC	*PacI*	DD-myc-F1 (R)
11	CCTGCAGGCCATGGCCAAAGTCGTCGCCAAGA AAAAGACCGAGTC	*SbfI, NcoI*	DD-myc-F1-ΔH (F)<br>His-F1-ΔH (F)
12	TTAATTAAGAATTCTCACTCACCGACTTTCTTG ACTGC	*PacI, EcoRI*	DD-myc-F1-ΔH (R)<br>His-F1-ΔH (R)
13	GCCAAGCTGCAAGAAATTGCGACAACAACGAA ACCCGTC	-	DD-myc-F1-IR/AA (F)<br>His-F1-R/A (F)
14	GACGGGTTTCGTTGTTGTCGCAATTTCTTGCAG CTTGGC	-	DD-myc-F1-IR/AA (R)<br>His-F1-R/A (R)
15	CGCGCATACAACATGAGCGCCGCCTTGTCCAA GTTCAAC	-	DD-myc-F1-IR/AA (F)<br>His-F1-IR/AA (F)
16	GTTGAACTTGGACAAGGCGGCGCTCATGTTGT ATGCGCG	-	DD-myc-F1-IR/AA (R)<br>His-F1-IR/AA (R)
17	ATGCATATGGGGGGTTCTCATCATCATCATC	*NsiI*	DD-myc-F2 (F)
18	TTAATTAATCAAGGAGAGGAAAGAGCGTCC	*PacI*	DD-myc-F2 (R)
19	CCATGGGAATGCATAAGAGCGACGAGGCTG CTGTGAAGG	*NcoI, NsiI*	DD-myc-F2-ΔH (F)<br>His-GST-F2-ΔH (F)
20	TTAATTAAGAATTCTCAAGGAGAGGAAAGA GCGTCC	*PacI, EcoRI*	DD-myc-F2-ΔH (R)<br>His-GST-F2-ΔH (R)
21	GAGAAGCTCATGGAGTTCGCCACAACGGTCG ACAAGTCG	-	DD-myc-F2-R/A (F)<br>His-F2-R/A (F)
22	CGACTTGTCGACCGTTGTGGCGAACTCCATGA GCTTCTC	-	DD-myc-F2-R/A (R)<br>His-F2-R/A (R)
23	CGAGCGCAAAACGTCGCCGCCGTCCTTGCCAG GCTTCCC	-	His-F2-IR/AA (F)
24	GGGAAGCCTGGCAAGGACGGCGGCGACGTTTT GCGCTCG	-	His-F2-IR/AA (R)
25	CCATGGGAAAGAAGCCGGAGGCACCCAAG	*NcoI*	His-F1L (F)
26	CCATGGGAAAGAAGAAGGGCGAAGGCGGTTTG	*NcoI*	His-F1 (F)
27	GAATTCTCACTCACCGACTTTCTTGACTGC	*EcoRI*	His-F1 (R)
28	CCATGGGAACCGGTTCGTATCCTGGTGCC	*NcoI*	His-F2 (F)
29	CCGGCATATGGGAGGCCTTAAGGTCGGCAAGA AGGACGAGG	*NdeI, StuI*	pET3amycHisF1 (F)
30	CGCGGATCCCTCCTTGCGTTTCAATTCGCG	*BamHI*	pET3amycHisF1 (R)
31	CCGGCATATGCTACGTAGAAAAATCCACTGGAA AACTCTCGGG	*NdeI*	pET3amycHisF2 (F)
32	CGCGGATCCCGCTTTACAGTTCAAGTAGCTTTCT CTC	*BamHI*	pET3amycHisF2 (R)
33	GGTACCGCCGAAAAAAGTGTTTTAGCAATTGTA CC	*KpnI*	pSD141/CtPbFRM1 (F)
34	GGGCCCAAATATTTTGAAAGATTTCTTTTCAGT	*ApaI*	pSD141/CtPbFRM1 (R)

	GTTTTCAACTG		
35	GGTACCGGTATTAAATTACATTGGCAATTATTA CCAACAC	*KpnI*	pSD141/CtPbFRM2 (F)
36	GGGCCCTTTTGATTTCTTTTCTTTAATGTCACCA TCATTAATTAAATG	*ApaI*	pSD141/CtPbFRM2 (R)
37	CAATACCCCCCAAAAATACACCTATTTTGG	-	CtPbFRM1 to check integration (F)
38	GCCACCACCACCCCTTCCAAAGTCAC	-	CtPbFRM2 to check integration (F)
39	AAATATATAAGTAAGAAAAACGGCGGCCG	-	Primer in the myc tag to check integration (R)
40	CGTGAGCCCGATCCGTACG	-	TgFRM1 annotation (F)
41	CCACGATGTACCGGAGCTCC	-	TgFRM1 annotation (R)
42	CGGACGCCATGCTCCAGG	-	TgFRM1 annotation (F)
43	CGGCTTGATGCTGCAGACACC	-	TgFRM1 annotation (R)
44	CCTGCCACTGGTCGACCC	-	TgFRM1 annotation (F)
45	CATATGTCTTGCCCGGCAAGCTGCC	-	TgFRM1 annotation (F)
46	CATATGCCCCGTCCAGCGTATCCG	-	TgFRM1 annotation (F)
47	GAGGCCTTCAGGAACTCCCGCTTTGGCTGCAG	-	TgFRM1 annotation (R)
48	GAGGCCTGCCGGAGGACCAGACC	-	TgFRM1 annotation (R)
49	CCGCCAGAGAATCTGCGCC	-	TgFRM1 annotation (R)
50	GTACAACCAACCGGACGACGC	-	TgFRM1 annotation (F)
51	CCTGCTGGTTCTTCCCTACGCG	-	TgFRM1 annotation (R)
52	GGCCGACTCACACGAGCTCGG	-	TgFRM1 annotation (F)
53	CGACCTCGAATGCACCAGCCG	-	TgFRM1 annotation (R)
54	GAGTGAGGAGACCAAGCAAGGG	-	TgFRM1 annotation (F)
55	GCTCAGAGCGAGGCTCTCG	-	TgFRM1 annotation (R)
56	GAAGGAGGCGCCAAAGAAGG	-	TgFRM1 annotation (F)
57	GCGCTTCCGGAAAGTCTCAG	-	TgFRM1 annotation (R)
58	ATGCTCACGAAAGAAGCGAGGAGC	-	TgFRM2 annotation (F)
59	GCACCTGAAGGGGAAGAATCTACAGC	-	TgFRM2 annotation (R)
60	GTGGAAGTATCGCCTCAAGAGG	-	TgFRM2 annotation (F)
61	CGGCGGAGGAAGCCACG	-	TgFRM2 annotation (R)
62	GCGTCCAATGCTGATAACCCC	-	TgFRM2 annotation (F)
63	CGTGAGCCCGATCCGTACG	-	TgFRM1 to check integration (F)
64	GAGAAACTGGCGTCGAAAGCCTTCG	-	TgFRM1, to check integration(R)
65	CGCAGTTCTCGGAAGACGCGTCGC	-	SAG4 promoter, to check integration (F)
66	CGTTTTGTTCCTGTCGGAACTAGGCG	-	TgFRM1, to check integration (F)
67	CCCAGCCGACGCGACAGCCTTC	-	NtTgFRM1, to check integration (R)
68	CCGGGCATGCAGGAGAAAAAAATCACTGGA	-	CAT to check integration (F)
69	GCCCCGCCCTGCCACTCATCGC	-	CAT to check integration (R)
70	GCTGGATCCGAGCAGAAGCTCATCTCCGAGGAG GACCTGGCTGCA	-	myc tag, to check integration (R)

Table S2: *T. gondii* strains used and generated in this study.

**Strain**	**Description**
RHΔhxgprt or RH	Virulent type 1 strain with disruption of HXGPRT gene.
TATi-1	RHΔhxgprt containing transcription transactivator-Tet repressor fusion under the control of Tubulin promoter.
mycTgFRM1-iKO	TATi-1 strain transformed with ptetO7Sag4mycTgFRM1 vector.
DD-F1	RH strain transformed with DD-myc-F1.
DD-F2	RH strain transformed with DD-myc-F2.
DD-F1-ΔH	RH strain transformed with DD-myc-F1-ΔH.
DD-F2-ΔH	RH strain transformed with DD-myc-F2-ΔH.
DD-F1-IR/AA	RH strain transformed with DD-myc-F1-IR/AA.
DD-F2-R/A	RH strain transformed with DD-myc-F2-R/A.
DD-F1-IR/AA/DD-F2-R/A	RH strain transformed with both DD-myc-F1-IR/AA and DD-myc-F2-R/A.

Supplementary figure legends:

Figure S1. (A) Experimental assembly of *TgFRM1* and *TgFRM2* genes. Diagram representing the *TgFRM1* and *TgFRM2* genes structures based on experimental annotations by RT-PCR, cloning and sequencing and on ToxoDB database predictions. Exons are represented as hatched (experimentally annotated) or as dark grey boxes (ToxoDB predictions) and are numbered as follows: Exon 1 to 7 (E1-E7). Introns are represented as black lines and are numbered as follows: Intron 1 to 6 (I1-I6). Arrowheads indicate the annotated start and stop codons of *TgFRM1* and *TgFRM2* genes. Dashed lines below the schemes represent the positions of experimentally annotated sequences. E.A: experimentally annotated. The scale bar represents 500 bps. **(B) FH2 domains alignment.** The formins 1 and 2 FH2 domains of Plasmodium and Toxoplasma have been aligned with the FH2 domain from yeast Bni1p formin, using CLUSTAL multiple alignment (http://npsa-pbil.ibcp.fr/cgi-bin/npsa_automat.pl?page=npsa_clustalw.html). The weakly similar residues are shown in blue, strongly similar residues are shown in green, and identical residues are in red. The asterisks indicated the W residue, which is conserved among all formin proteins, and the two residues I and R or K that have been shown to be critical for actin binding and are conserved in all described formins. The amino acid numbers for the initial residues of the expressed recombinant proteins are shown in parentheses on the left. **(C-D) Generation of selective antibodies recognizing TgFRM1 and TgFRM2.** Antibodies raised against the *E. coli* produced FH2 domains of TgFRM1 (**panel C**) and TgFRM2 (**panel D**) specifically recognize the corresponding FH2 domains in transgenic parasites. DD: destabilization domain fused to TgFRM1 and 2 FH2 domains. Asterisks indicate degradation products. RHΔhxgprt represents the wild type strain. DD-myc-F1 corresponds to the transgenic strain expressing wild type formin 1 FH2 domain fused to the destabilization domain of FKBP

(DD). DD-myc-F2 corresponds to the transgenic strain expressing wild type formin 2 FH2 domain fused to DD.

Figure S2. (A-B) TgFRM1 conditional KO by promoter exchange strategy. (A) Schematic representation of the strategy used to replace the endogenous promoter of TgFRM1 with the tetracycline inducible promoter. The ptetO7Sag4mycNtTgFRM1 plasmid contains 3000 bps of the 5' flanking region of *TgFRM1* (in black), the chloramphenicol acetyl transferase (CAT) gene (in blue) controlled by tubulin promoter (*TUB5*) (blue arrow) and the myc-tagged (in red) N-terminal coding sequence of *TgFRM1* (in grey, 1500 bps) under the control of the inducible tetO7Sag4 promoter (orange arrow). The YFP-YFP cassette is represented in green. Black arrows represent the primers used for PCR analysis and the length of the PCR fragments generated is indicated. **(B)** PCR analysis performed on mycTgFRM1-iKO, showing that double homologous recombination occurred. Genomic DNA from TATi-1 parasites was used as negative control. **(C) Inducible expression of TgFRM1 in mycTgFRM1-iKO by IFA.** Down regulation of mycTgFRM1i in the mycTgFRM1-iKO strain as shown by IFA with anti-myc antibodies 96 hrs after ATc treatment. Scale bars represent 2 μm.

Figure S3. Epitope tagging of PbFRM1 and PbFRM2 by knock-in strategy. (A) Insertion of a myc epitope tag at the C-terminus of *P. berghei* FRM1 and FRM2 by single homologous recombination (knock-in). The position of the PCR primers used for genotyping the transgenic parasites are indicated by numbered arrows. **(B-C, left panels) PCR analysis of the myc-tagged locus.** Genomic DNA isolated from pool of pyrimethamine-resistant parasites transfected with either pSD141/CtPbFRM1 or pSD141/CtPbFRM2 (left) or from wild-type parasites (right) was subjected to PCR using the primers indicated (Lanes 1 and 3, primers 1 + 2 (diagnostic for the wild-type locus); lanes 2 and 4, primers 3 + 4 (diagnostic for 3′ integration). The transfected

parasites show integration of the plasmids, and the wild-type locus is detectable in these parasites. **(B-C, right panels) Western blot of myc-tagged PbFRM1 and PbFRM2.** Parasites extracts were fractionated by SDS-PAGE, transferred to a Hybond membrane and probed with an HRP-conjugated anti-myc polyclonal antibody. Lanes 1: untransfected parasites; lanes 2: parasites transfected with either pSD141/CtPbFRM1 or pSD141/CtPbFRM2**.** The size of PbFRM1 is about 210 kDa, and about 350 kDa for PbFRM2. WT: wild-type parasites, KI: Knock-in.

Figure S4. (A-C) Schematic representation of the FH2 domains expressed in bacteria as fusion with His-tag (in the pETHTB vector), or downstream of a His-GST-tag (in the pETM30 vector). F1L (469 amino acids (aa), 4582-5051): wild-type FRM1 FH2 domain plus 48 amino acids, F1 (421 aa, 4630-5051): wild-type FRM1 FH2 domain, F1-R/A (421 aa, 4630-5051): mutated FRM1 FH2 domain (the R4867 residue is mutated into A residue), F1-IR/AA (421 aa, 4630-5051): mutated FRM1 FH2 domain (the I4713 and R4867 residues are mutated into A residue), F1-ΔH (367 aa, 4684-5051): truncated FRM1 FH2 domain (the N-terminal part encompassing the two helices responsible for dimerization is deleted), F2 (727 aa, 3317-4043): wild-type FRM2 FH2 domain including the potential FH1 domain (enriched in glycine, lysine, and proline residues), F2-R/A (727 aa, 3317-4043): mutated FRM2 FH2 domain (the R3709 residue is mutated into A residue), F2-IR/AA (727 aa, 3317-4043): mutated FRM2 FH2 domain (the I3511 and R3709 residues are mutated into A residue), F2-ΔH (564 aa, 3480-4043): truncated FRM2 FH2 domain (the N-terminal part encompassing the first two helices responsible for dimerization as well as the potential FH1 domain are deleted). Asterisks indicate the mutated residues either I (left) or R (right). Numbers refer amino acids positions in the entire protein sequence. **(B-D)** Diagrammatic representation of the TgFRM1 and 2 FH2 domains (the same as in A and C) cloned into *T. gondii* vectors downstream of the destabilization domain and a myc tag (in the pTUB8DDmyc vector).

Asterisks indicate the mutated residues either I (left) or R (right). Numbers refer amino acids positions in the entire protein sequence.

Figure S5. (A-B) Purification of recombinant FH2 domains of TgFRM1 and TgFRM2. Separation of His tagged FH2 domains expressed in *E. coli* (BL21) and purified on nickel columns. Proteins were loaded on SDS page 10% followed by Coomassie blue staining. Asterisks indicate bacteria histidine rich proteins or chaperone binding to Nickel beads and are eluted with the truncated formin 1 FH2 domain under native conditions. **(C-D) Oligomerization of bacterially produced His-F1L and His-F2 *in vitro*. (C)** Exclusion chromatography of F1L and F2 were analyzed using a Superose 6 10/300 GL column. The chromatograms display the absorbance at 280 nm as a function of elution volume. Two peaks were observed with both FH2 domains indicating that they can dimerize or oligomerize. The reference standard was bovine serum albumin (BSA) (67 kDa). **(D)** Eluate fractions analysis. The presence of specific proteins in the eluate fractions was checked by Coomassie (F1L) or by western blot using anti-TgFRM2 antibodies (F2). Asterisk indicates degradation product. **(E, F, G) Assessment of the biochemical properties of TgFRM1 and TgFRM2 in binding to TgACT1 *in vitro* and *in vivo*. (E)** Nickel affinity pull-down of *T. gondii* actin (TgACT1) from mycTgPRF expressing parasite lysates with recombinant His-GST-F1, His-GST-F1L, and His-GST as negative control. Western blots were revealed using anti-actin, anti-myc antibodies and anti-catalase antibodies as negative control. The FT, W1, and W2 are added to show the amount of actin in the different fractions of the experiment. FT: flow through (detection of parasite actin unbound to the bait protein after incubation), W1 and W2 correspond to the first and second wash. **(F)** Nickel affinity pull-down of *T. gondii* actin (TgACT1) from mycTgPRF expressing parasite lysates with recombinant His-GST-F2 and His-GST used as negative control. **(G)** TgACT1 association with TgFRM1 and TgFRM2 as determined by co-IP with total parasite lysates. TgFRM2 and mycTgFRM1i were

immunoprecipitated using rabbit anti-FRM2 and mouse anti-myc antibodies respectively and resolved by western blot analysis. Preimmune serum (PI) corresponding to the anti-FRM2 antibody was used as negative control. An extract of RH parasites was used as negative control for the mycTgFRM1-iKO strain. Samples were subjected to immunoblotting with the mAb anti-actin, or with the anti-FRM1 and anti-FRM2 specific antibodies. Heavy (HC) and light (LC) chains of the mouse immunoglobulins are detected with the mouse anti-actin antibodies. **(H) Effect of F2-R/A and IR/AA mutations on barbed end disassembly.** The experiment was conducted as in Fig. 3H. Filaments were depolymerized in the absence (black) and in the presence of 99 nM F2 (red), 99 nM F2-R/A (green) and 99 nM F2-IR/AA (magenta).

Figure S6. (A) Controlled expression of the series of FH2 domains by Shld-1. (A) Western blot analysis of parasites grown in presence and absence of Shld-1 for 48 hours using anti-myc antibodies. **(B)** Immunofluorescence assays performed on transgenic parasites expressing the different DD-mycFH2 WT or mutant domains of TgFRM1 and 2 using anti-myc antibodies. Parasites were grown in presence of Shld-1 for 24 hours before fixation and staining with the indicated antibodies. Scale bars represent 2 µm. **(C-D) F1 or F1-IR/AA or F2 or F2-R/A but not F1-ΔH or F2-ΔH form heterodimers with their respective formins.** Rabbit antisera raised against TgFRM1 **(C)** and TgFRM2 **(D)** recognize full-length formins as well as wild-type, mutated, and truncated FH2 domains on western blots. Extracellular parasites from all described transgenic parasites were lysed in a buffer containing 0.2% TX100, and subjected to IP with the myc antibodies. Proteins bound to the beads were separated by SDS-PAGE and analyzed by western blots using anti-TgFRM1 and anti-TgFRM2 antibodies, respectively. White asterisks represent DD-F1 and DD-F1-IR/AA degradations. Blue asterisks indicate IgG heavy chains. Parasite total lysates (T), immunoprecipitated proteins eluted from beads (IP).

Figure S7. (A, B, C) Expression of wild type F1 and F2 but not F1-ΔH or F2-ΔH affects parasite growth. (A) Plaque assays were performed on parasite strains expressing F1, F2, F1-ΔH, or F2-ΔH. HHF monolayers were infected with parasites in presence or in absence of Shld-1, fixed after 6 days, and stained with Giemsa. The scale bar represents 1 mm. **(B)** Intracellular growth assay of indicated parasite strains (RHΔhxgprt, DD-F1). Replication was analyzed in presence or in absence of Shld-1 after 63 hrs of treatment. Values are means ± SD for three independent experiments. **(C)** Intracellular growth assay of indicated parasite strains (RHΔhxgprt, DD-F2). Replication was analyzed in presence or in absence of Shld-1 after 63 hrs of treatment. Values are means ± SD for three independent experiments. **(D) Intracellular growth analysis of parasites expressing DD-F1-IR/AA or DD-F2-R/A or DD-F1-IR/AA plus DD-F2-R/A.** Intracellular growth assay of indicated parasite strains (RHΔhxgprt, DD-F1-IR/AA, DD-F2-R/A, and DD-F1-IR/AA/DD-F2-R/A). Replication was analyzed in presence or in absence of Shld-1 after 63 hrs of treatment. Values are means ± SD for three independent experiments.

Video S1 Live circular gliding video at 5X speed of DD-F2-R/A parasites none treated with Shld-1.

Video S2 Live abortive circular gliding video at 5X speed of DD-F1-IR/AA parasites treated with Shld-1.

Video S3 Live abortive circular gliding video at 5X speed of DD-F2-R/A parasites treated with Shld-1.

Video S4 Live helical gliding video at 5X speed of DD-F1-IR/AA parasites none treated with Shld-1.

Video S5 Live abortive helical gliding video at 5X speed of DD-F1-IR/AA parasites treated with Shld-1.

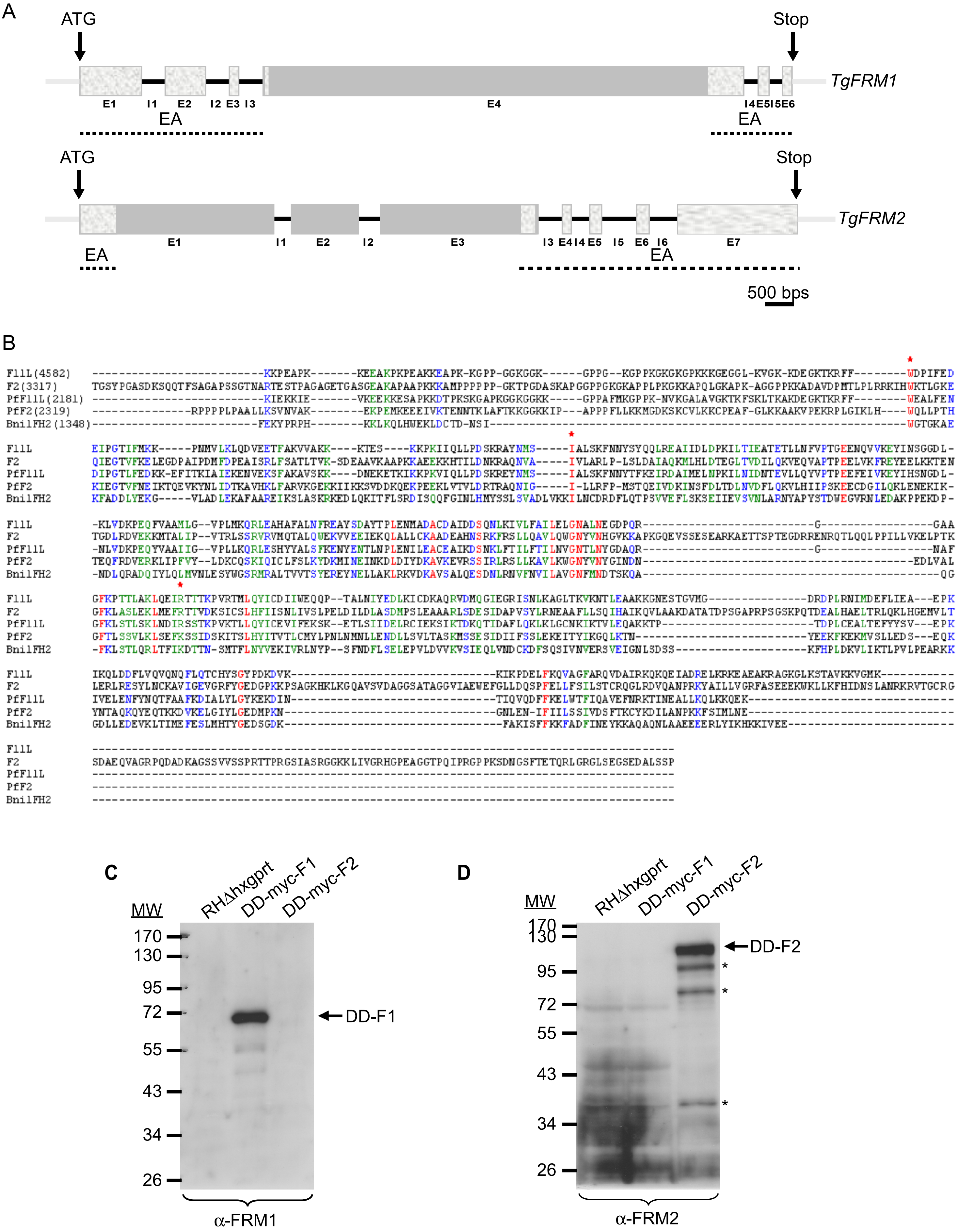

A
ATG
Stop
TgFRM1
E1
I1
E2
I2
E3
I3
E4
I4
E5
I5
E6
EA
EA
ATG
Stop
TgFRM2
E1
I1
E2
I2
E3
I3
E4
I4
E5
I5
E6
I6
E7
EA
EA
500 bps
B
F1L(4582)
F2(3317)
PfF1L(2181)
PfF2(2319)
BnilFH2(1348)
C
MW
RHΔhxgprt
DD-myc-F1
DD-myc-F2
170
130
95
72
55
43
34
26
DD-F1
α-FRM1
D
MW
RHΔhxgprt
DD-myc-F1
DD-myc-F2
170
130
95
72
55
43
34
26
DD-F2
*
*
*
α-FRM2

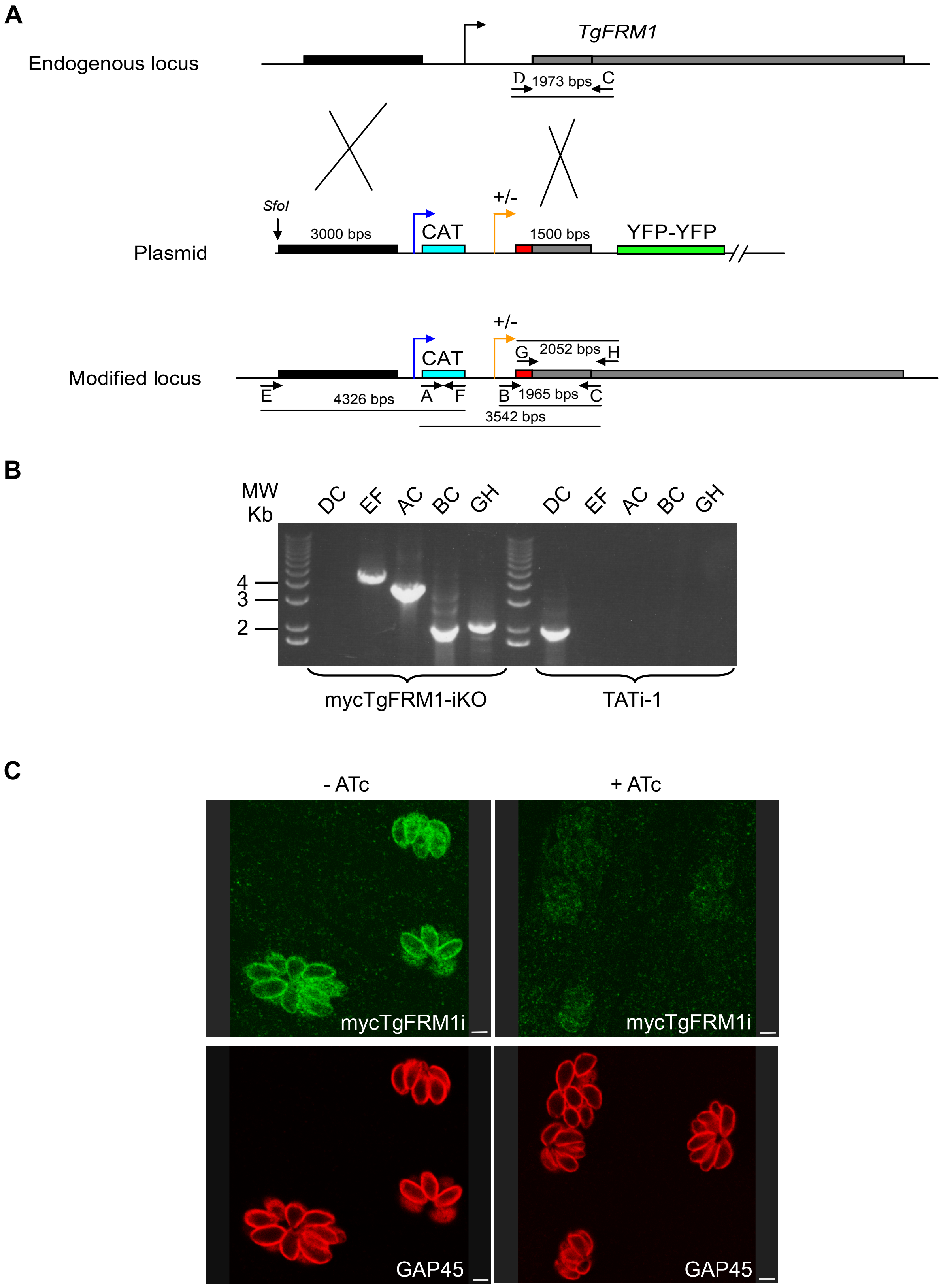
A
TgFRM1
Endogenous locus
D
1973 bps
C
SfoI
+/-
3000 bps
CAT
1500 bps
YFP-YFP
Plasmid
+/-
CAT
G
2052 bps
H
Modified locus
E
4326 bps
A
F
B
1965 bps
C
3542 bps
B
MW
Kb
DC
EF
AC
BC
GH
DC
EF
AC
BC
GH
4
3
2
mycTgFRM1-iKO
TATi-1
C
- ATc
+ ATc
mycTgFRM1i
mycTgFRM1i
GAP45
GAP45

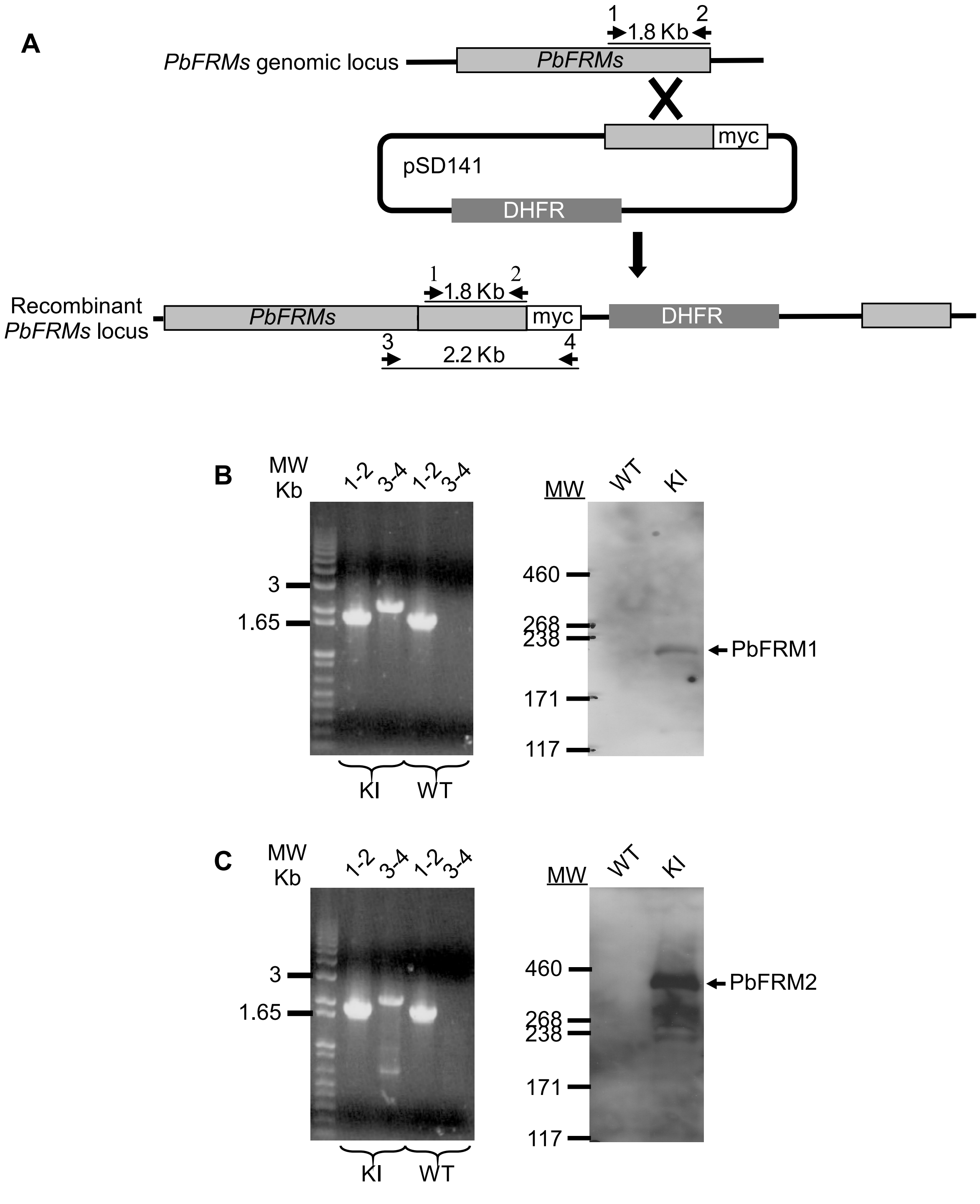

A
PbFRMs genomic locus
PbFRMs
1
2
1.8 Kb
myc
pSD141
DHFR
Recombinant
PbFRMs locus
PbFRMs
1
2
1.8 Kb
myc
DHFR
3
4
2.2 Kb
B
MW
Kb
1-2
3-4
1-2
3-4
3
1.65
KI
WT
MW
WT
KI
460
268
238
171
117
PbFRM1
C
MW
Kb
1-2
3-4
1-2
3-4
3
1.65
KI
WT
MW
WT
KI
460
268
238
171
117
PbFRM2

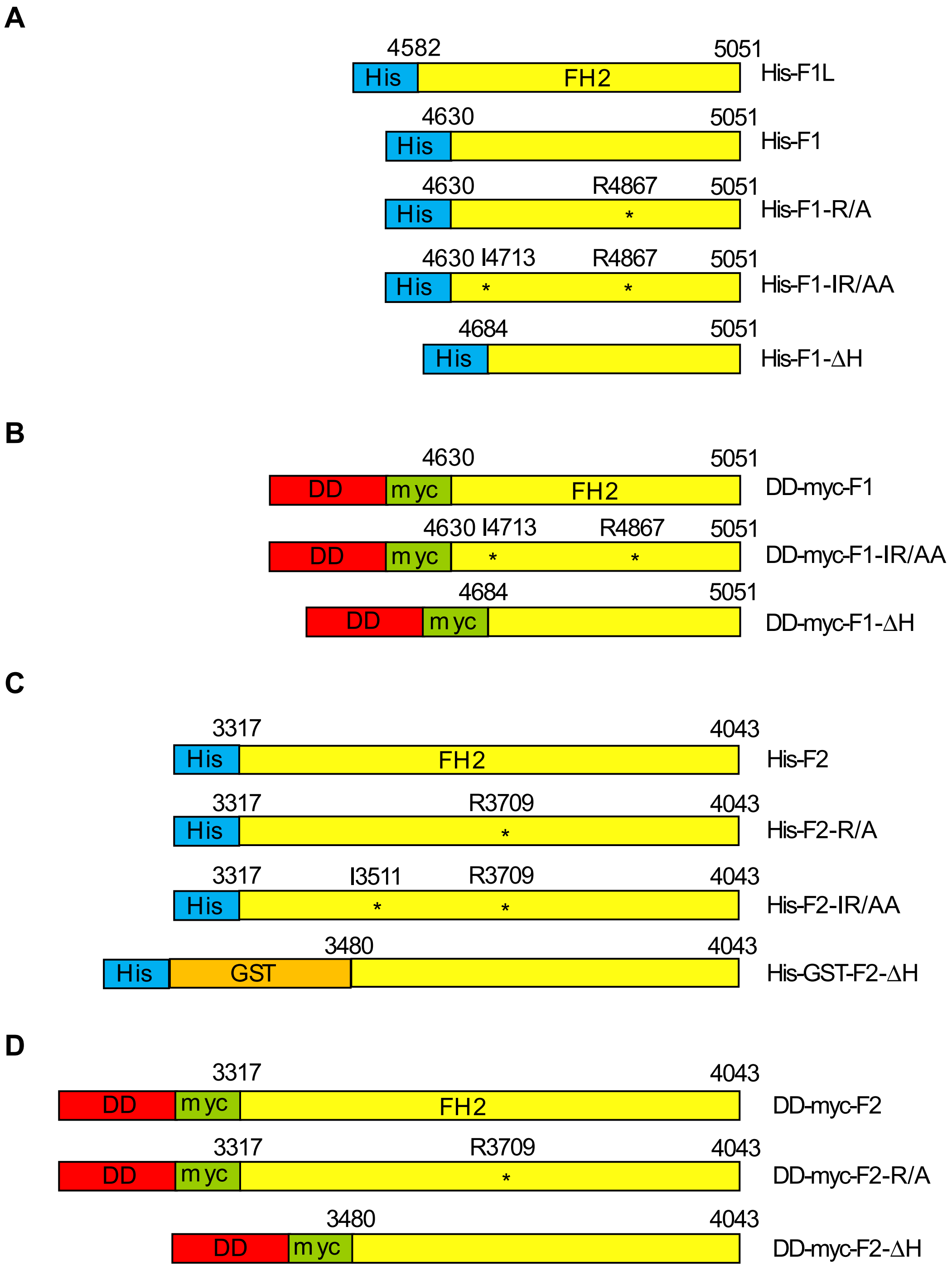

A
4582
5051
His
FH2
His-F1L
4630
5051
His
His-F1
4630
R4867
5051
His
*
His-F1-R/A
4630 I4713
R4867
5051
His
*
*
His-F1-IR/AA
4684
5051
His
His-F1-ΔH
B
4630
5051
DD
myc
FH2
DD-myc-F1
4630 I4713
R4867
5051
DD
myc
*
*
DD-myc-F1-IR/AA
4684
5051
DD
myc
DD-myc-F1-ΔH
C
3317
4043
His
FH2
His-F2
3317
R3709
4043
His
*
His-F2-R/A
3317
I3511
R3709
4043
His
*
*
His-F2-IR/AA
3480
4043
His
GST
His-GST-F2-ΔH
D
3317
4043
DD
myc
FH2
DD-myc-F2
3317
R3709
4043
DD
myc
*
DD-myc-F2-R/A
3480
4043
DD
myc
DD-myc-F2-ΔH

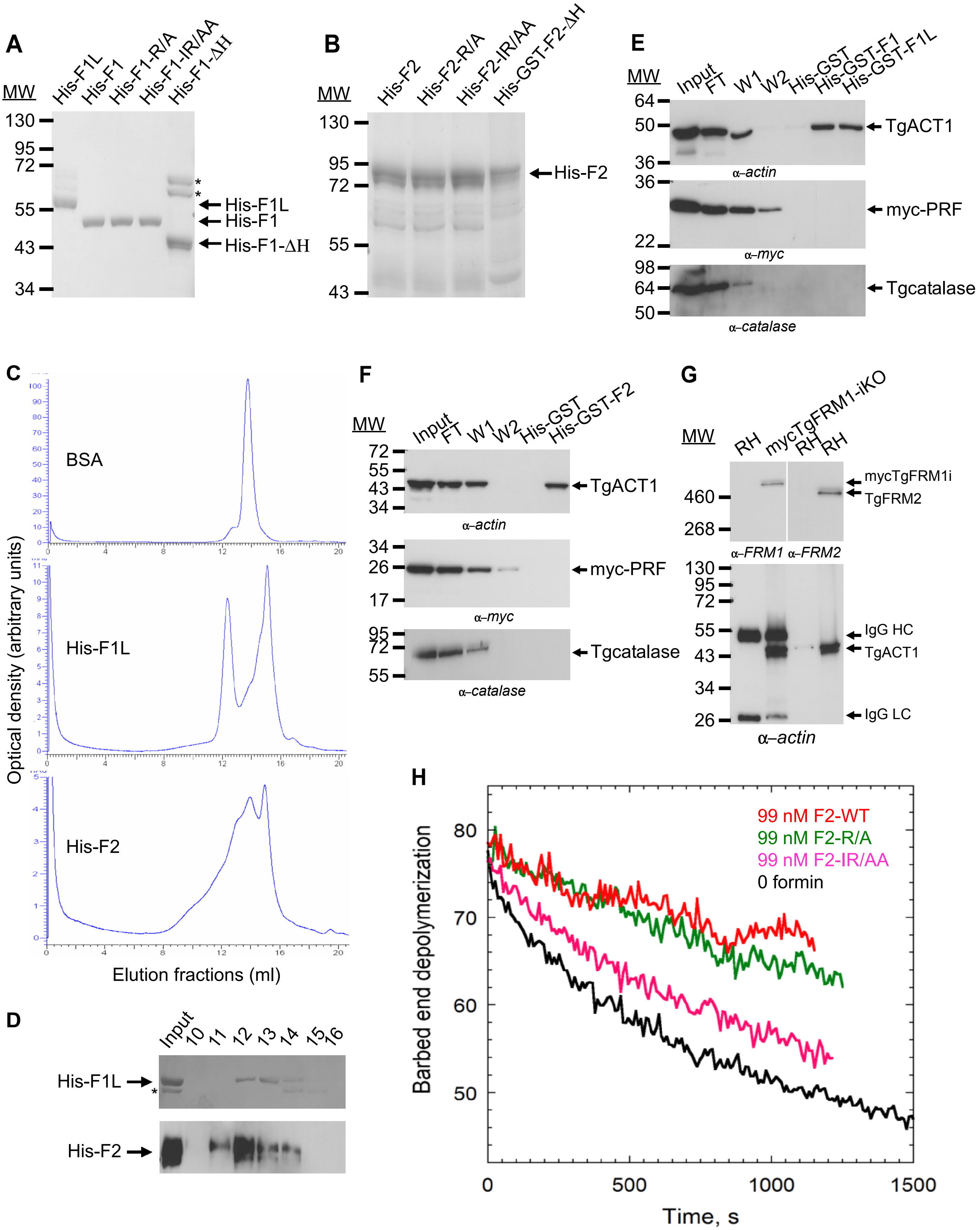
A
MW
His-F1L
His-F1
His-F1-R/A
His-F1-IR/AA
His-F1-ΔH
130
95
72
55
43
34
His-F1L
His-F1
His-F1-ΔH
B
MW
His-F2
His-F2-R/A
His-F2-IR/AA
His-GST-F2-ΔH
130
95
72
55
43
His-F2
E
MW
Input
FT
W1
W2
His-GST
His-GST-F1
His-GST-F1L
64
50
36
TgACT1
α–actin
36
22
myc-PRF
α–myc
98
64
50
Tgcatalase
α–catalase
C
BSA
His-F1L
His-F2
Optical density (arbitrary units)
Elution fractions (ml)
F
MW
Input
FT
W1
W2
His-GST
His-GST-F2
72
55
43
34
TgACT1
α–actin
34
26
17
myc-PRF
α–myc
95
72
55
Tgcatalase
α–catalase
G
MW
RH
mycTgFRM1-iKO
RH
RH
460
268
mycTgFRM1i
TgFRM2
α–FRM1
α–FRM2
130
95
72
55
43
34
26
IgG HC
TgACT1
IgG LC
α–actin
D
Input
10
11
12
13
14
15
16
His-F1L
His-F2
H
99 nM F2-WT
99 nM F2-R/A
99 nM F2-IR/AA
0 formin
Barbed end depolymerization
80
70
60
50
0
500
1000
1500
Time, s

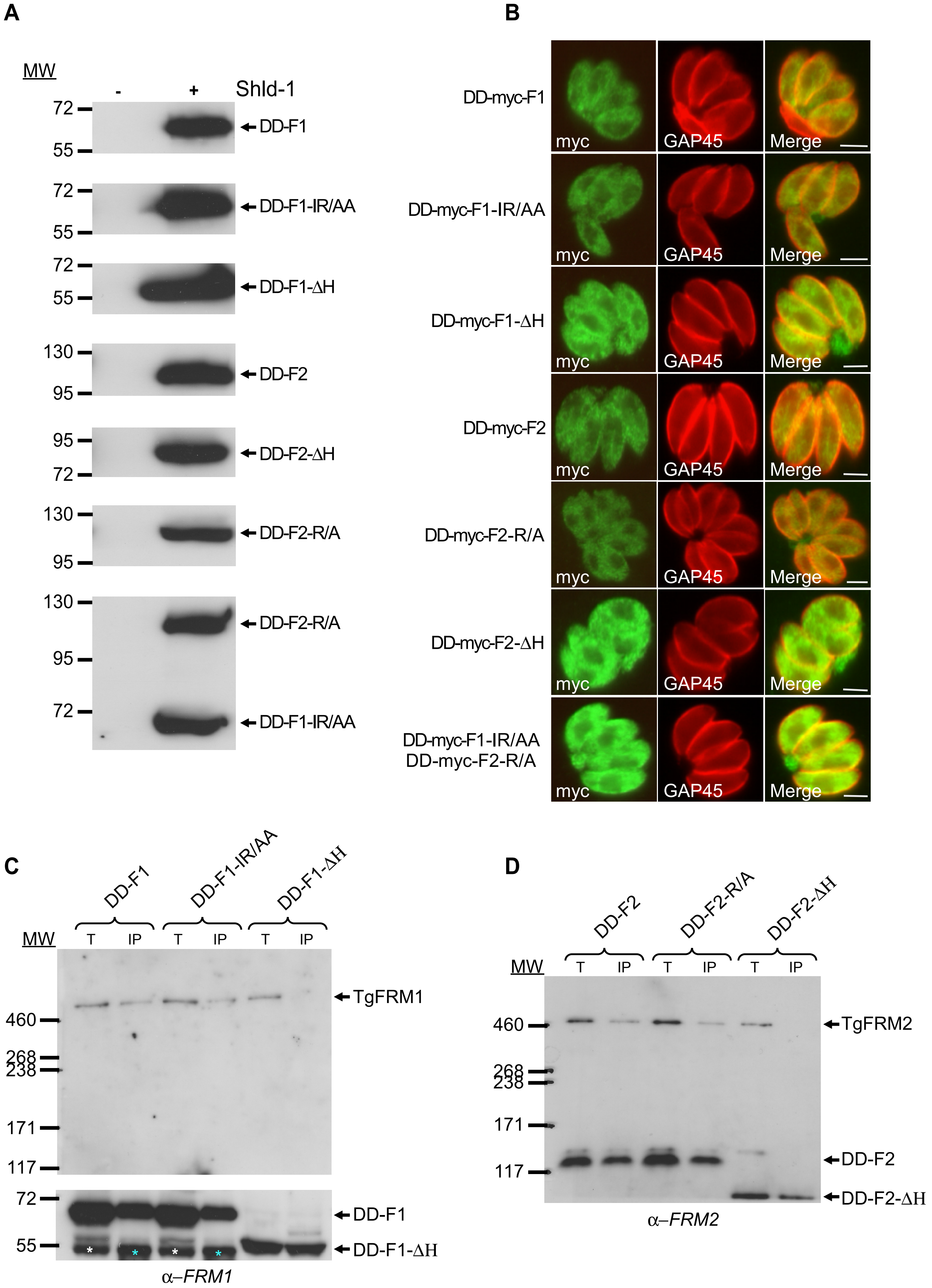
A
MW
-
+
Shld-1
72
55
DD-F1
DD-F1-IR/AA
DD-F1-ΔH
130
95
DD-F2
DD-F2-ΔH
DD-F2-R/A
B
DD-myc-F1
DD-myc-F1-IR/AA
DD-myc-F1-ΔH
DD-myc-F2
DD-myc-F2-R/A
DD-myc-F2-ΔH
DD-myc-F1-IR/AA
DD-myc-F2-R/A
myc
GAP45
Merge
C
DD-F1
DD-F1-IR/AA
DD-F1-ΔH
T
IP
460
268
238
171
117
TgFRM1
DD-F1
DD-F1-ΔH
α–FRM1
D
DD-F2
DD-F2-R/A
DD-F2-ΔH
TgFRM2
DD-F2
DD-F2-ΔH
α–FRM2

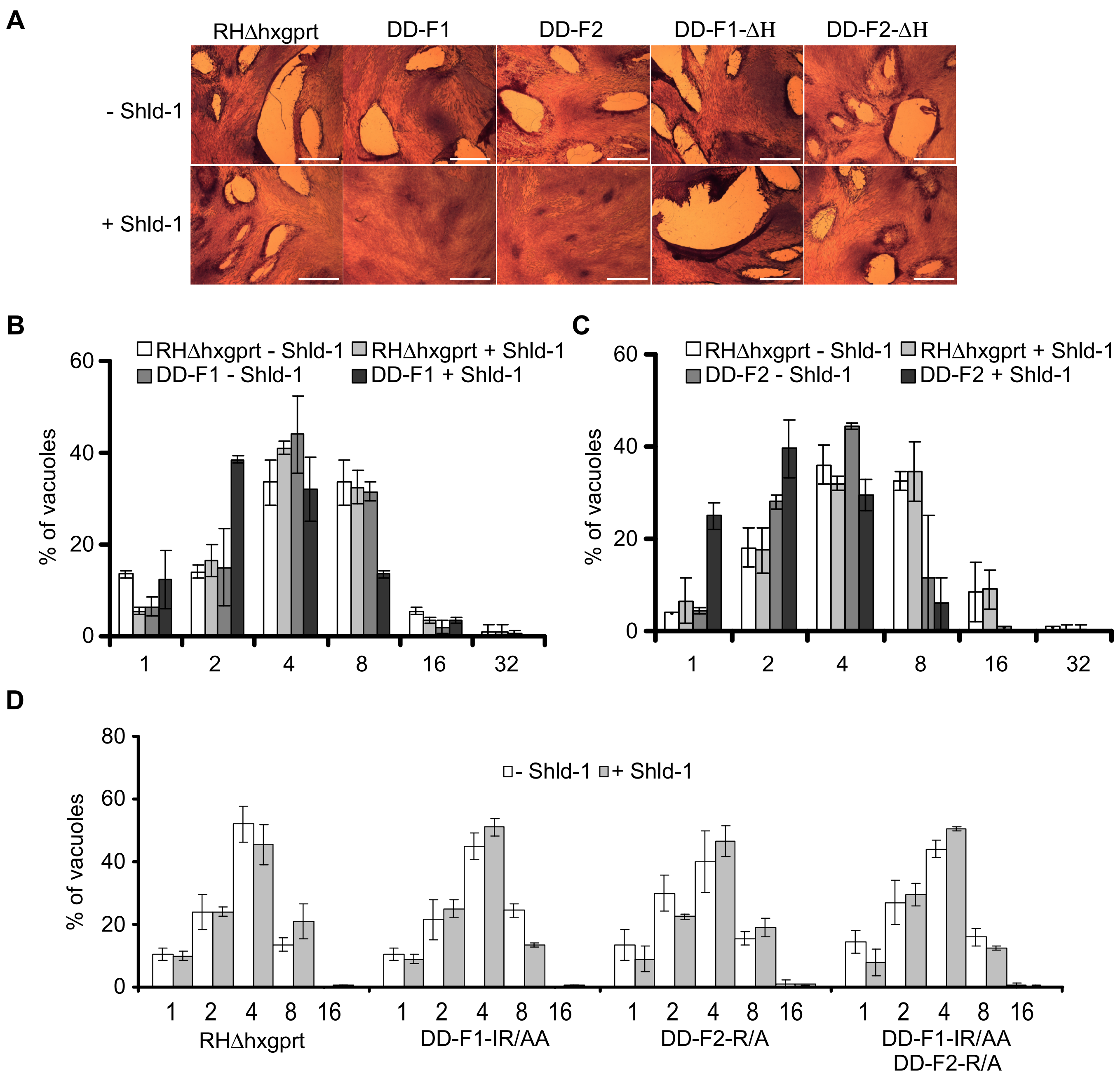
A
RHΔhxgprt
DD-F1
DD-F2
DD-F1-ΔH
DD-F2-ΔH
- Shld-1
+ Shld-1
B
RHΔhxgprt - Shld-1
RHΔhxgprt + Shld-1
DD-F1 - Shld-1
DD-F1 + Shld-1
% of vacuoles
0
20
40
60
1
2
4
8
16
32
C
RHΔhxgprt - Shld-1
RHΔhxgprt + Shld-1
DD-F2 - Shld-1
DD-F2 + Shld-1
% of vacuoles
0
20
40
60
1
2
4
8
16
32
D
- Shld-1
+ Shld-1
% of vacuoles
0
20
40
60
80
1 2 4 8 16
RHΔhxgprt
1 2 4 8 16
DD-F1-IR/AA
1 2 4 8 16
DD-F2-R/A
1 2 4 8 16
DD-F1-IR/AA
DD-F2-R/A
